# Radiometal chelators for infection diagnostics

**DOI:** 10.3389/fnume.2022.1058388

**Published:** 2023-01-09

**Authors:** Asma Akter, Oliver Lyons, Varun Mehra, Heather Isenman, Vincenzo Abbate

**Affiliations:** 1 Department of Analytical, Environmental and Forensic Sciences, King’s College London, London, United Kingdom; 2 Vascular Endovascular and Transplant Surgery, Christchurch Public Hospital, Christchurch, New Zealand; 3 Department of Surgery, University of Otago, Christchurch, New Zealand; 4 Department of Hematology, King’s College Hospital NHS Foundation Trust, London, United Kingdom; 5 Department of Infectious Diseases, General Medicine, Christchurch Hospital, Christchurch, New Zealand

**Keywords:** infection diagnostics, radiometals, gallium-68, siderophores, chelators, PET/CT imaging, immunocompromised

## Abstract

Infection of native tissues or implanted devices is common, but clinical diagnosis is frequently difficult and currently available noninvasive tests perform poorly. Immunocompromised individuals (for example transplant recipients, or those with cancer) are at increased risk. No imaging test in clinical use can specifically identify infection, or accurately differentiate bacterial from fungal infections. Commonly used [^18^F]fluorodeoxyglucose (18FDG) positron emission computed tomography (PET/CT) is sensitive for infection, but limited by poor specificity because increased glucose uptake may also indicate inflammation or malignancy. Furthermore, this tracer provides no indication of the type of infective agent (bacterial, fungal, or parasitic). Imaging tools that directly and specifically target microbial pathogens are highly desirable to improve noninvasive infection diagnosis and localization. A growing field of research is exploring the utility of radiometals and their chelators (siderophores), which are small molecules that bind radiometals and form a stable complex allowing sequestration by microbes. This radiometal-chelator complex can be directed to a specific microbial target *in vivo*, facilitating anatomical localization by PET or single photon emission computed tomography. Additionally, bifunctional chelators can further conjugate therapeutic molecules (e.g., peptides, antibiotics, antibodies) while still bound to desired radiometals, combining specific imaging with highly targeted antimicrobial therapy. These novel therapeutics may prove a useful complement to the armamentarium in the global fight against antimicrobial resistance. This review will highlight current state of infection imaging diagnostics and their limitations, strategies to develop infection-specific diagnostics, recent advances in radiometal-based chelators for microbial infection imaging, challenges, and future directions to improve targeted diagnostics and/or therapeutics.

## 1 Introduction

**Figure 1:**
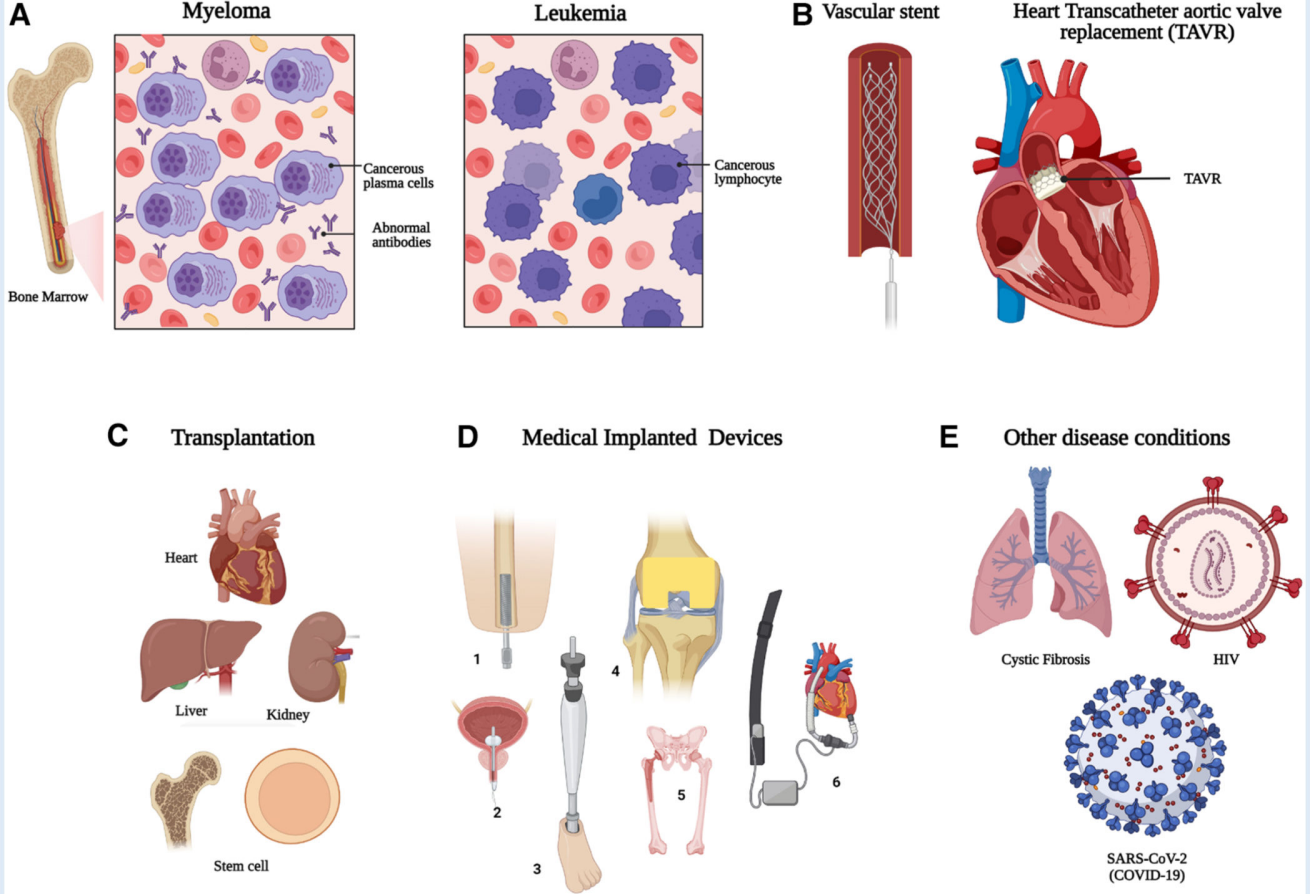
Individuals with increased risk of opportunistic and invasive microbial infection which require early and specific diagnostics. (**A**) Hematological malignancy, (**B**) vascular stents and grafts, (**C**) organ and stem cell transplantation, (**D**) Other medical implants devices/prostheses, (**1** = Bone implant; **2** = Urinary catheter; **3** = Prosthetic leg; **4** = Knee implant; **5** = Hip replacement; **6** = Ventricular assisted device) and (**E**) other example diseases conditions with increased risk of prosthetic infection. HIV: Human Immunodeficiency Virus; SARS-CoV-2: Severe Acute Respiratory Syndrome Coronavirus 2. Created with BioRender.com.

Microbial infections pose major threats to global human health and clinical practice. These threats may appear as a viral pandemic or increasing antimicrobial resistance in bacterial and fungal pathogens (1). The ongoing SARS-CoV-2 pandemic shows how vulnerable we are to evolving pathogens and effort is needed to detect and treat microbial infections early (2). Moreover, individuals with underlying health conditions for example heart disease, diabetes, surgical implantations/prostheses, organ or stem cell transplantation, cancer or other forms of immunodeficiency states (hereditary or acquired) are at greater risk of comorbidity and mortality (Figure 1). Global populations are ageing, and infection and comorbidity are more common in the elderly, and are expected to become an ever increasing clinical problem (3).

Although antimicrobials have been one of the most successful forms of chemotherapy in the last century for bacterial and fungal infections (4), bacterial infections remain a significant healthcare challenge worldwide, mainly due to the ancient yet ongoing evolution and transmission of Antimicrobial Resistance (AMR) genes (5–7). It is estimated that by 2050, AMR-related mortality may reach >10 million per year, which is comparable to that from cancer (8). A recent systematic analysis by a team of collaborators working on global AMR revealed that about 1.27 million deaths were directly attributable to AMR in 2019 (9). The more prevalent resistant pathogens are *Escherichia coli, Staphylococcus aureus, Klebsiella pneumoniae, Streptococcus pneumoniae, Acinetobacter baumannii, and Pseudomonas aeruginosa*. On the other hand, the global burden of Invasive Fungal Infections (IFI) is less well established, but may lead to a “silent crisis” with over 150 million cases of IFI estimated annually worldwide, resulting in approximately 1.7 million annual deaths (10). Among the fungal pathogens, *Aspergillus fumigatus, Candida albicans*, and other Candida species (including *C. auris*) are particularly adept in causing life-threatening IFI.

To address this global crisis, a multifaceted approach is required where new or re-purposed antimicrobial candidates are urgently developed, and more sensitive and specific noninvasive rapid diagnostics put in place to guide appropriate choice of antimicrobials and to monitor treatment efficacy. Since few novel antimicrobial candidates are in the development pipeline (11), it is becoming increasingly important to rationalize drug use to only those with a confident diagnosis of an infection, and for the shortest effective duration required (12).

Existing laboratory diagnosis of microbial infections involves microbiological, serological and molecular techniques, where biological samples are collected from patients, often in an invasive manner, and analyzed in a laboratory (13). Traditional microbial culture techniques incubate these samples in the lab using various growth media to confirm the presence of specific microbial isolates and perform antibiotic resistance profiling to inform the choice of antibiotic treatment. These results may require further confirmation by serological (using antibodies specific to pathogens) and other molecular analytical techniques like 16S/18S-ribosomal RNA identification and gene sequencing, or Matrix-Assisted Laser Desorption/Ionization-Time Of Flight (MALDI-TOF) Mass Spectrometry (MS) (14–16). Several of these novel approaches do not require culture in a laboratory (17). These approaches are time consuming and immediate results are often not available. Additionally, they lack real-time monitoring capabilities to determine the overall burden of the disease, or identify the anatomical site of infection. Universal imaging techniques like plain radiographs, magnetic resonance imaging (MRI), ultrasound scan (US) and computed tomography (CT) are being used as first line imaging techniques for detection of signs of inflammation, for example fluid accumulation or abscess formation, as a surrogate marker of infection (18, 19). However, none of these techniques can accurately discriminate between infectious and non-infectious (sterile) inflammation, nor identify microbial pathogens.

Imaging in nuclear medicine uses a small amount of a radioactive tracer injected intravenously, combined with detection of emitted radiation to provide anatomical localization. Radioactive tracers are radiopharmaceuticals based on radionuclides, radiometals and/or their chelators. Chelators bound to radiometals can sequester specific radionuclides/radiometals inside the body. In general, radiometals with suitable chelators are linked with a biological targeting unit (with or without a spacer/linker), which will bind to the specific biomolecule. When a non-metallic radionuclide is used, chelators are omitted, and the radionuclide is linked with the targeting unit by a covalent bond producing a radiopharmaceutical. Commonly, in nuclear medicine either a positron emission tomography (PET) or single photon emission computed tomography (SPECT) technique is utilized to observe metabolic and functional processes, assess cellular function and to diagnose and treat disease. A combined modality/multimodal imaging technique is suitable for obtaining both anatomical and molecular attributes, to provide enough information for clinical diagnosis of a disease. Therefore, PET/CT, SPECT/CT or PET/MRI multimodalities are more desirable for developing infection specific imaging. The most widely used combined modality for infection imaging—PET/CT using [^18F^]fluorodeoxyglucose (18FDG)—is based on localizing a host’s inflammatory immune response rather than imaging the pathogen itself. It has substantial limitations as 18FDG PET/CT detects high glucose uptake in tissues (e.g., the brain), but does not discriminate accurately between infection and sterile inflammation, and cannot identify the infecting pathogen (20–22).

Radiometals technetium-99m (^99m^Tc), indium-111 (^111^In) and gallium-67 (^67^Ga) have mostly been used for clinical infection diagnostics. In common clinical practice, human leukocytes are labeled with ^99m^Tc and ^111^In to localize inflammation as in indication of infection. They have been used in tuberculosis (TB), infective endocarditis (IE), osteomyelitis, Fever of Unknown Origin (FUO), and inflammatory diseases (23). [^67^Ga]Ga-citrate (using citrate as the metal chelator) has been used to localize infection in osteomyelitis, lung infections, FUO, etc (23). However, like 18FDG, all these imaging techniques lack specificity, and produce signal in a sterile inflammatory process or malignancy (24, 25).

In recent years, the utility of radiometal gallium-68 (^68^Ga)-based molecular imaging has grown considerably. ^68^Ga has a short half-life of ~68 min compared to 72 h for ^67^Ga, and it is readily produced from commercial ^68^Ge/^68^Ga generators in widespread clinical use compared to ^67^Ga, which requires a cyclotron. Also, ^67^Ga requires SPECT imaging, whereas ^68^Ga is imaged *via* PET. These advantages facilitate use of ^68^Ga-based radiotracers for imaging cancer and infections. Various chelators for ^68^Ga have already been investigated to advance cancer diagnosis (26–28). Among the radiometals and Bifunctional Chelators (BFCs) developed for the detection of pathogens, specifically [^68^Ga]Ga-1,4,7-Triazacyclononane-1,4,7-triacetic acid (NOTA)-ubiquicidin (UBI) is noteworthy (29, 30). UBI is an antimicrobial peptide produced by the human immunological system. Another BFC, 2,2',2",2"’-(1,4,7,10-tetraazacyclododecane-1,4,7,10-tetrayl) tetraacetic acid (DOTA) was radiolabeled with ^68^Ga and conjugated with the peptide UBI to detect *S. aureus in vitro* and *in vivo* in a small-animal infection model (31). Preclinical studies were not accompanied with any other bacteria or fungal strain to investigate its spectrum of detection. Nevertheless, this approach has demonstrated the potential of radiolabeled antimicrobial peptides to detect pathogens by PET/CT imaging.

To develop useful infection-specific PET or SPECT radiotracers, direct localization of pathogens (for example to a particular organ, or the surface of an implant) is critical. They may have the capability of differentiating bacterial infection from fungal infections and specifying different bacterial or fungal species. More recently, targeting a specific mechanism in microbial pathogens with siderophores (iron-chelating small molecules produced by microorganisms and plants) has been investigated to evaluate the potential of these siderophores for imaging microbial infections. Siderophore-based infection imaging in conjunction with PET/CT has shown great preclinical prospects in terms of both broad- and narrow-spectrum infection tracer development. Siderophores, being high-affinity iron chelators, can be used to chelate ^67^/^68^Ga due the chemical similarity between iron and gallium. This review will mainly focus on the current status and future direction of siderophore-based infection imaging tracer development while discussing other approaches to develop noninvasive diagnostics of microbial infection in high-risk immunocompromised patients.

## 2 Targeted patient groups for developing infection diagnostics

### 2.1 Infection in cancer patients

While cancer itself may impair individuals’ immunity, cancer treatments like chemotherapy, radiotherapy as well as immunosppressive treatments like bone marrow stem cell transplants and targeted biological drugs can significantly impair immunity and render individuals prone to microbial infection. For example, people with cancer can suffer from severe leucopenia, where the white blood cell count becomes very low due to radiation or chemotherapy treatment (32). Consequently, this lowers the natural immunity to fight against a range of infections, in particular bacterial and fungal pathogens. These infections may be mild, or severe enough to be life threatening, for example, mild skin infection or a fatal fungal pneumonia. Patients with life-threatening blood cancers (e.g., acute leukemia/high grade lymphoma or advanced myeloma) may require more aggressive chemotherapeutic treatment, resulting in greater risk of developing fungal infections (33). Moreover, novel targeted drugs and biological immunotherapies including CAR-T (Chimeric antigen re-engineered T cells) are changing the risk profile for developing serious opportunistic infections. Significant challenges remain in terms of early detection, management, and monitoring success of therapeutic interventions for these infections.

In terms of fungal risk following treatment related immunosuppression, patients with haematological malignancies mostly suffer from invasive aspergillosis (primarily caused by *A. fumigatus*) and some develop invasive candidiasis (mainly caused by *C. albicans*). Invasive aspergillosis in high-risk immunocompromised patients has an attributable mortality of 27%–72% making it a leading cause of death in this group of patients (34). Invasive candidiasis is one of the most common blood stream infections in Intensive Care Units (ICUs) and the associated mortality rate is 20%–30% (35). Less common Candida species like *C. parapsolosis* and *C. glabrata* are contributing to an increasing proportion of infections, with rising rates of azole (a commonly used class of antifungal agent) resistance (36). These IFI remain difficult to diagnose in a timely fashion, as there is no universally available “gold standard” test. Routine microbiological tests with fungal culture often do not yield positive isolates or lead to false negative results and thus may fail to determine the overall burden of the disease. Diagnosis based on fungal biomarkers including beta-D-glucan and galactomannan, which lack specificity, combined with radiological features and histological changes (EORTCMSG criteria) are helpful but imprecise (37, 38). They may still underestimate the incidence of IFI, specifically with use of newer antifungal prophylaxis regimens that reduce the diagnostic sensitivity and specificities of these methods.

Audits in UK hemato-oncology units have shown an incidence of proven IFI of 6.5%–21%, while up to 50% of patients get treated with empirical antifungals during febrile neutropenia episodes (39). Thus, many of these patients may still receive antifungal treatment despite no IFI due to poor diagnostic tools, and this potential overuse of antifungals may lead to serious consequences for individual patients and potentially drive antifungal resistance. In addition, it has an economic impact on healthcare systems. The National Health Service of England’s antifungal budget is approximately £150M and is increasing year on year, with the majority (£87M) spent in haemato-oncology units (39). Globally, antifungal resistance is spreading rapidly, including the emergence of new Multidrug Resistant (MDR) fungal pathogens associated with increased mortality. Additionally, reports of increasing azole-resistant infections caused by *A. fumigatus*—the commonest cause of invasive aspergillosis in the UK, and rapid spread of the MDR organism, *C. auris*, are of significant clinical concern (40, 41). Improvement requires early sensitive and specific detection of fungal infections with effective noninvasive diagnostic tools for IFI, which remains an unmet need.

### 2.2 Vascular graft and endograft infection

Prosthetic vascular tube grafts (placed at open surgery) and endovascular stents or endografts are increasingly used to treat life-threatening arterial aneurysms, dissection, and occlusive vascular disease (42). Vascular Graft Infection (VGI) is estimated to complicate 0.5%–6% implanted grafts depending on the anatomical location of the implant, causing significant morbidity, mortality, and economic burden (43, 44). Vascular implant infection is more common when the device has been used to treat infective pathology of the blood vessel itself, for example a mycotic aneurysm. Depending on the location of the graft, up to 70% mortality with VGI is reported, and graft infections in the lower limbs frequently lead to amputation. No arterial graft is designed to ever be removed, and doing so mandates major surgery to remove the graft and then reconstruct the blood supply to downstream tissues (45). The economic burden associated with VGI is £541M per year in the United States alone (46). These costs may increase due to the widespread use of aortic endografts in elderly and co-morbid patients, in whom infection is more common. The morbidity associated with other vascular implants such as intravenous access devices, pacemakers and internal cardiac defibrillators remains a major clinical challenge since treatment options are poor. The current focus remains on prevention (47, 48).

A very wide range of organisms are implicated in vascular infection, and the most common organisms tend to vary by the site of the implant and the presence of an associated graft-enteric or graft-respiratory tract fistula. The most frequently isolated bacteria are *S. aureus* (30%) and *S. epidermidis* (17%) (49). Although less common, graft infections due to Gram-negative bacteria have increased in frequency. *Enterococcus faecalis* is the predominant pathogen in cases of aorto-enteric fistulae (50). While acute infections occur within four months following the surgery and are associated with virulent bacteria, late/chronic infections can occur many years of surgery, for example following a bacteremia. The causative agents of mycotic aneurysms vary by geographical location, for example salmonella infection is more common in Asia. The diagnosis of VGI is well-recognized to be challenging, and again as there is no “gold standard test” for the condition, it is instead defined by syndromic criteria (47, 51). Laboratory findings may be helpful (raised inflammatory markers, and positive blood cultures), but are neither sensitive nor specific. Current clinically available imaging techniques usually provide non-specific information and cannot identify causative pathogens (beyond the identification of visceral fistulae, indicating likely polymicrobial infection). As almost all patients are already receiving antimicrobial treatment at the time of surgical removal of the implant, microbiological identification is difficult as bacterial cultures often remain negative, rendering targeted treatment impossible unless an organism is identifiable by non-culture based techniques (e.g., PCR) (52). Use of 18FDG is limited by the tendency of prosthetic implants to drive a sterile inflammatory reaction (53), which can be hard to differentiate from low-grade inflammation caused by infection. It is notable that many decades after the identification of mycotic aneurysms as a pathological entity, there remains no accepted consensus diagnostic criteria, and the imaging features remain woefully non-specific. The problem is complicated by the identification of bacteria in approximately one third of aneurysms that would otherwise not be considered “mycotic”, in mural thrombus, and in atheromatous plaque (54, 55). The ability to diagnose the presence of pathogens within the blood vessel wall, or within atheroma, would represent a major clinical advance (56).

### 2.3 Individuals with solid organ transplants

Immunosuppressive drugs play a major role in the prevalence of infections after transplantation of solid organs (kidney, liver, heart, intestine, and lung), which may lead to the recipient’s death within the first year of transplantation (57, 58). A Swiss Transplant Cohort Study (SOTS) between 2008 and 2014 with ≥12 months of follow-up, revealed 55% patients developing >3,500 infection episodes during their first-year post transplantation (59). Among these infections, bacterial infections were most prevalent (63%), predominantly Gram-negative Enterobacteriace in heart, lung, kidney and liver transplant recipients, specifically *P. aeruginosa* and Gram-positive *Enterococcus* spp. About 30% were viral infections associated with kidney, liver and heart transplant recipients. Although fungal infections were least common (~7.5%), *Candida* spp. was mostly prevalent (60%) in liver transplant patients, while *A. fumigatus* (1.4%) infection was lowest in all solid organ transplant recipients. The diagnostic uncertainty and delays in diagnosis remains a challenge in these immunocompromised patients, often leading to graft rejection and high mortality risk.

### 2.4 Infection in other medically implanted devices

With the advancement of medical science and technology, patients are often treated with temporary or permanent implantable devices including dental, cardiac, orthopedic implants and prostheses. These devices pose a risk of becoming infected, especially in immunocompromised patients or those with other underlying health conditions. According to the United States Centre for Disease Control (CDC), 50%–70% of the 2 million healthcare-associated or nosocomial infections could be attributed to implanted medical devices (44). As expected, infections in all intravascular medical devices (including VGI) including cardiac pacemakers, mechanical heart valves, and ventricular assist devices, are identified as life-threatening (60). A high risk of mortality (>25%) is associated with prosthetic valve endocarditis (61). Ventilator-associated pneumonia in intubated patients is one of the most common infections in the ICU (62). The most common bacteria found in intravascular devices and prosthetic joints are coagulase-negative staphylococci (CoNS) (e.g., *S. epidermidis*), then *S. aureus*, and less common pathogens include *Enterococcus* spp., *Candida* spp., *E. coli* and *Klebsiella* species (63). Conversely, *E. coli*, *Candida* spp., and *Enterococcus* spp. are the most common in urinary catheter related infections (64). Along with nosocomial pathogens, opportunistic microorganisms from the host’s respiratory, urinary, and gastrointestinal tract can also colonize the implanted device.

### 2.5 Other immunosuppressed states

Outside of cancer, those with diabetes, cirrhotic liver disease, receiving steroid therapy or living with untreated HIV, cystic fibrosis (CF), chronic granulomatous disease as well as those treated for severe COVID-19 disease, are among the most significant groups of immunocompromised patients, who are at high risk of serious microbial infections.

Pulmonary infections are a major cause of morbidity and mortality in people living with untreated HIV, including with *Strep*. pneumoniae, in particular due to *Pneumocystis* spp., and *Mycobacterium tuberculosis* (TB). Worldwide, roughly one third of people living with HIV/AIDS become co-infected with TB (65). In 2015, there were 1.14 million new cases of tuberculosis in people with HIV/AIDS, of which 400,000 people died as a direct result of TB (66). Death due to TB is more common in low- and middle-income countries, in particular Africa, which has higher rates of MDR TB (66). Patients with untreated HIV have a greater risk of progression from latent to active TB, with an associated increased risk of onward nosocomial TB transmission. In addition, people living with untreated HIV are susceptible to a myriad of fungal infections, including cryptococcal disease, mucocutaneous candidiasis, and aspergillosis (67).

CF, one of the most common lethal genetic disorders in Caucasians, affects over 60,000 patients globally (68). Patients living with CF commonly develop pulmonary infections with *P. aeruginosa*. Other microorganisms include *S. aureus*, *M. abscessus*, *Burkholderia cepacia*, and *A. fumigatus*. Emerging MDR *P. aeruginosa* and *S. aureus* infection in CF patients are a growing concern, and preclude these patients from consideration of lung transplantation (69, 70).

The recent COVID-19 pandemic also revealed the comorbidity and co-mortality in patients from bacterial or fungal infection in those with severe SARS-CoV-2 infection (71). Most reported fungal infections in COVID-19 patients include aspergillosis, candidiasis, and mucormycosis. The main causative agent for mucormycosis is *Mucorales* spp., often incorrectly called a “black fungus infection” (72). Fungal infections resistant to antifungal treatment have also been described in patients with severe COVID-19 (73), but diagnostic uncertainties remain with lack of specific and sensitive biomarkers or imaging tools.

Similarly, causative agents for FUO (74), osteomyelitis and septic arthritis (75) as well as deep soft tissue and deep wound infections (76) are sometimes difficult to confirm, and so empirical therapy is often used. Table 1 summarizes the pathogens requiring particularly urgent development of specific infection imaging diagnostics.

## 3 Current status and future prospects: radiotracers for infection

### 3.1 Radionuclides and chelators in nuclear medicine

**Figure 2:**
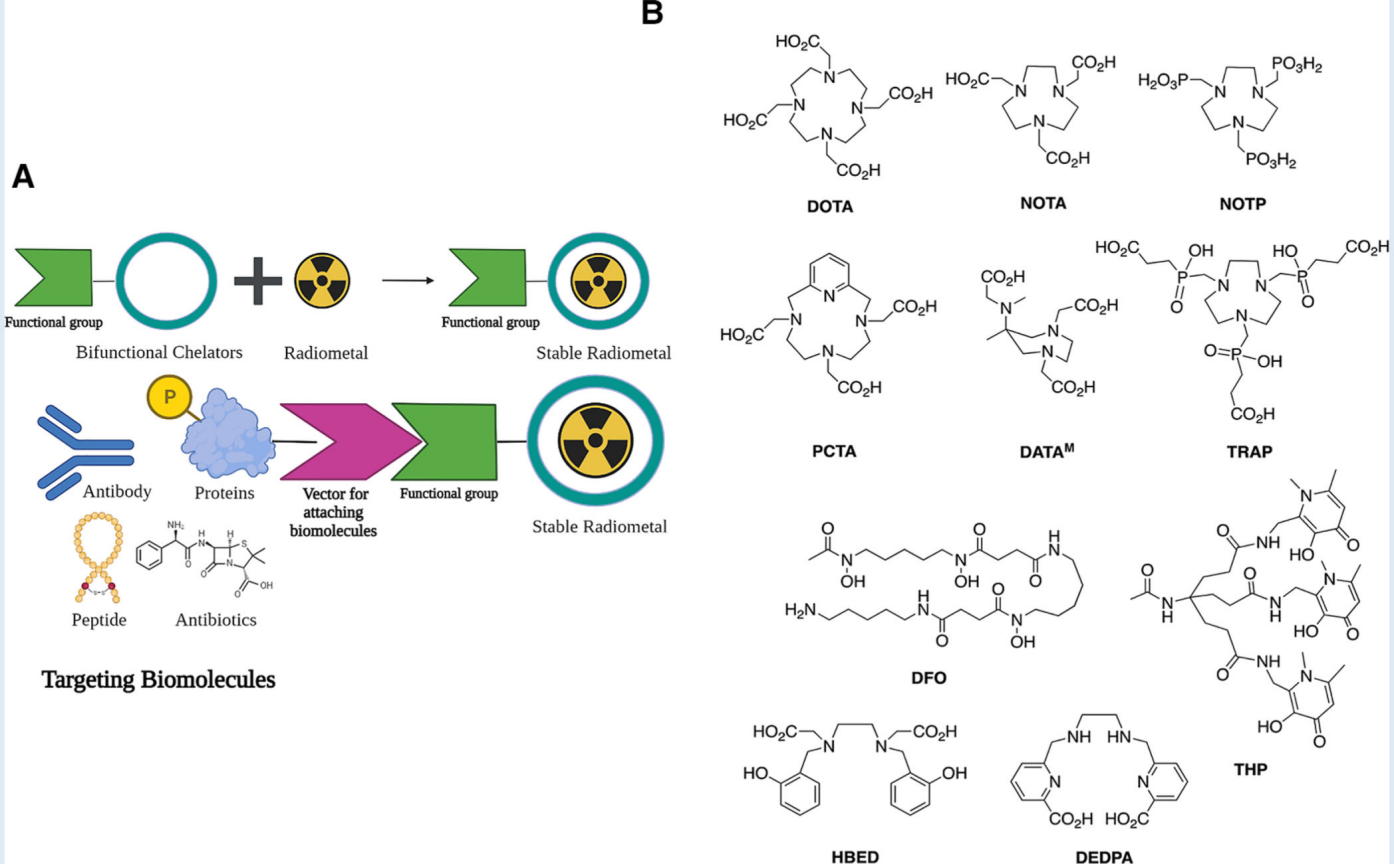
(**A**) Bifunctional chelators (**BFCs**) and targeting biomolecules for diagnostic and therapeutic purposes. Created with BioRender.com. (**B**) BFCs for gallium-68. Reproduced from Ref. (23) with permission from the Royal Society of Chemistry under Creative Commons Attribution 3.0 Unported Licence. Reproduced with BioRender.com.

The potential arsenal of radionuclides (both metal and non-metal) in nuclear medicine is rich, and both PET and SPECT imaging radiometals are available for evaluating organ function, detecting cancer, measuring blood flow, and investigating metabolic processes. The major advantage of using nuclear medicine over other imaging techniques is to achieve detailed functional and anatomical (combined with CT/MRI) changes through the administration of radioactive compounds to patients, in very low dose (nanomolar or less) which is otherwise difficult to obtain. An ideal radiometal to be used in nuclear medicine must fulfil the first requirement of coordinated complex stability, both during radiolabeling and in the *in vivo* system. This stability can be achieved by binding the radiometal with a ligand or chelator. The type of chelator used depends on the chemistry and application of the radiometals. This stable metal-ligand complex can then be administered in vivo, and PET/SPECT scanners can image the accumulation in different organs in the body. BFCs can complex metals and can be conjugated with targeting moieties (Figure 2A). This exerts specificity of the radiotracer for a particular disease diagnosis and possible treatment (theranostics). Therefore, the selection of radiometals and the design of BFCs are critical to develop diagnostics for any specific disease condition. Of note, a single BFC, which can accommodate different radiometals and biomolecules, would be preferable. Available radiometals and chelators currently used in nuclear medicine are summarized in Table 2. Figure 2B shows the structure of the most commonly used BFCs in nuclear medicine (77). Details of radiometals and their chelator chemistry have been discussed elsewhere (78, 79).

For imaging infection diagnostics, radiometals should meet some additional criteria:

(a) SPECT/PET imaging modality compatibility (preferably PET imaging as PET is easier to quantify than SPECT).
(b) Ease of production from a readily available clinical generator.
(c) No emission of damaging particles like alpha (α) or beta (β).
(d) Well known chelation chemistry and available BFCs.
(e) Short half-life of radiometals to reduce the overall radiation burden in patients, but sufficient half-life to allow imaging at the time when the infected cells/tissues to normal cells/tissues ratio reaches maximum [the optimum time required to prepare the dose and administer to patients and perform PET/CT scanning requires minimum of >3hours (85), therefore the desired half-life is 1–14 h]
(f) Low or no uptake in other non-desirable major organs or tissues in the body.
(g) Rapid clearance from the body (to reduce prolonged retention in other off-target organs).

**Figure 3:**
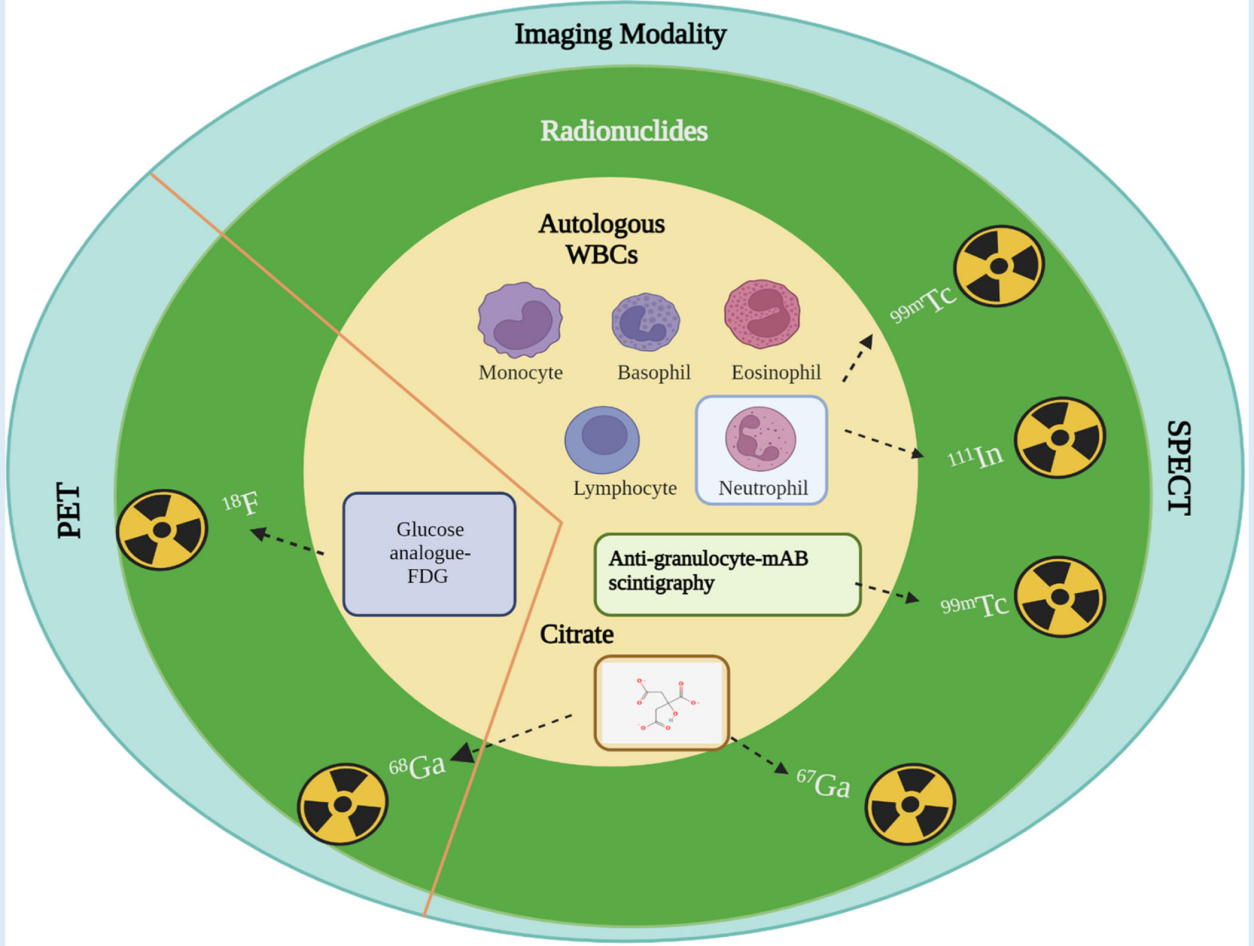
Mechanism of action of radionuclides for infection imaging in clinical practice. Autologous WBCs: Specific chemotactic activation; Anti-granulocyte: Increased capillary antibodies permeability and specific binding or uptake as antibody labelled granulocytes; Citrate: Transferrin and lactoferrin receptor binding; FDG: Upregulated glucose transporter-1 (GLUT-1) in activated granulocytes, lymphocytes, and monocytes. Created with BioRender.com.

Due to their short half-life and generator production, ^99m^Tc and ^68^Ga have been the suitable choice for current infection imaging. Most clinical diagnostics are based on ^99m^Tc, in part due to the low-cost of SPECT scanning. The mechanisms of commonly used radiopharmaceuticals for infection imaging are depicted in Figure 3, where white blood cells (WBCs) and anti-granulocyte monoclonal antibodies are labeled with ^99m^Tc, and cellular immune responses (the local accumulation of leucocytes) are observed. ^111^In is also utilized to label autologous WBC and chemotactic activation of WBCs in the body localize the site of the response to possible infection or inflammation. WBC labeling for infection imaging which can be done with [^111^In] In-oxine or with [^99m^Tc]Tc-exametazime/HMPAO. 18FDG is the most commonly used diagnostic PET radiotracer for clinical oncology and infectious diseases. FDG is simply the analogue of glucose and is labeled with nonmetal positron emitter ^18^F. When administered in patients, it shows increased uptake in normal and abnormal tissues with a high metabolic rate (due to infection, inflammation, abnormal cell growth) compared to background tissues. Although these imaging techniques lack proper specificity of pathogen detection, they are recommended in the diagnostic workup of infections. For VGI, the current gold standard in European Society of Vascular Surgery guidelines is 18FDG PET/CT or SPECT due to the better sensitivity and specificity (86, 87). Table 3 summarizes use of current infection diagnostics, their application and limitation.

### 3.2 Current strategies to develop microbial specific radiotracers

**Figure 4:**
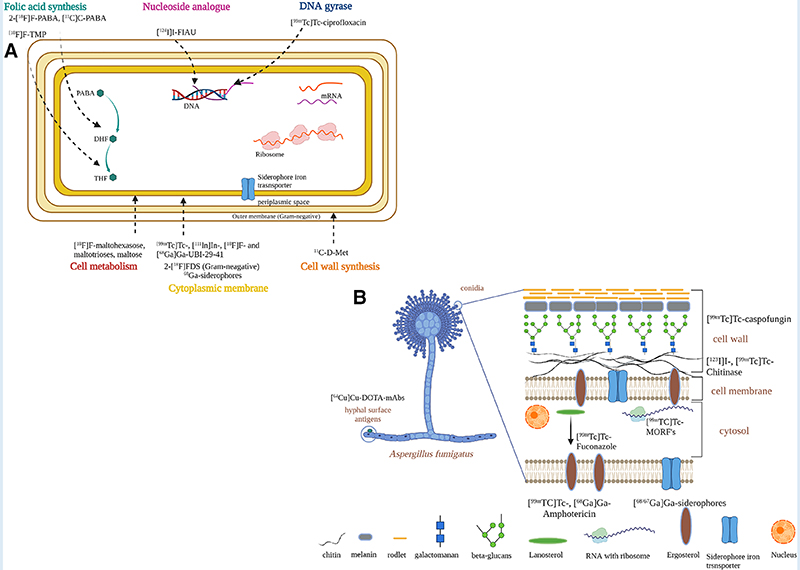
Strategies to develop infection-specific radiotracers for infection diagnostics. (**A**) bacteria-specific strategies. (**B**) fungal-specific strategies. Created with BioRender.com.

Development of diagnostics relies on careful exploration of differential biochemistry of the infective pathogen and its host. The current strategies to design infection-specific diagnostics are based on targeting microbial metabolic pathways or microbial cellular components, using antimicrobial peptides and antibiotics, and targeting essential or transitional metals for pathogens. Figure 4(A), 4(B) depicts the diagnostic developments specifically in bacterial and fungal cells, respectively.

#### 3.2.1 Different microbial metabolic pathways

Although *in vitro* study with 18FDG has confirmed uptake of 18FDG in clinically relevant Gram-positive and Gram-negative bacteria (105), non-specific *in vivo* accumulation of 18FDG in sterile inflammation and tumor tissues has limited its use as an infection-specific tracer (20–22).

Sorbitol analogue 2-[^18^F]fluorodeoxysorbitol (2-[^18^F]FDS) is metabolized selectively by Enterobacterales and has been evaluated in a small study using PET/CT before and after antibiotic use (106, 107). 2-[^18^F]FDS is specific to this group of bacteria and can be selective when there is a mix of pathogens but cannot differentiate each bacterial genus within the group. Further preclinical and clinical studies are needed in a range of infection models with Enterobacterales.

Non-metal ^18^F conjugated with other polysaccharides such as 6-[^18^F]fluoromaltose (108) and 6″-[18F]F-maltotriose (109) which target the maltodextrin transport pathway were investigated in both Gram-positive and Gram-negative bacteria. Preclinical studies in an *in vitro* and *in vivo* myositis model with *E. coli*, and a wound infection model with P. aeruginosa, showed promising results by distinguishing infection from sterile inflammation. Another study evaluated the potential of [^18^F]F-maltohexaose (18FMH) which also targeted the maltodextrin pathway in bacteria (110). In an in vivo myositis model *E. coli*, 18FMH showed higher sensitivity and specificity compared to 18FDG. 18FMH was able to detect early infection with a microbial load as low as 10^5^ Colony Forming Unit (CFU), and also drug-resistant *E. coli*. Although these tracers have shown promising results *in vivo*, the serum stability of these radiotracers could be comprised by the presence of starch-degrading enzymes in blood (111). To understand the structural requirement for high serum stability, synthesis of different ^18^F-labeled maltodextrins and their stability tests were performed (111). This study demonstrated that maltotriose is the optimum scaffold to develop a maltodextrin pathway-specific radiotracer, but preclinical small-animal infection models should consider higher starch-degrading enzyme activity than those observed in human blood. Another consideration is that these radiotracers will detect only metabolically active bacteria, and so might not detect bacteria in a quiescent state in a biofilm.

The Folic acid de-novo synthesis pathway in bacteria (which is not present in humans) has also been investigated to develop [^11^C]C-Para-aminobenzoic acid ([^11^C]C-PABA) (112), and 2-[^18^F]Fluoro-Para-aminobenzoic acid (2-[^18^F]F-PABA) (113) as substrates for folic acid synthesis, and preclinical evaluation (*in vitro* and small-animal *in vivo S. aureus* infection model by PET imaging or radioautography) showed the ability to distinguish between sterile inflammation and infection.

#### 3.2.2 Microbial specific cellular components

Bacteria-specific cellular components such as the cell wall precursor D-methionine (D-met) is absent in mammals. D-[methyl-^11^C]methionine (^11^C-D-Met) was developed in a preclinical animal model, where it was successful in distinguishing between *E. coli* and *S. aureus* infection from sterile inflammation (114). Likewise, Carbon-11 labeled D-alanine (D-ala) (due to the abundance of D-alanine in bacterial peptidoglycan) derivatives were synthesized and evaluated for their *in vitro* and *in vivo* characteristics for microbial infection detection (115). Both radiotracers D-[3–^11^C]alanine and the dipeptide D-[3–^11^C]alanyl-d-alanine were able to accumulate in bacterial cells in vitro. Particularly D-[3-^11^C]alanine showed uptake in both rodent models of discitis/osteomyelitis and *P. aeruginosa* pneumonia. Therefore, the study concluded that D-[3-^11^C]alanine has potential for clinical translation to diagnose clinically relevant bacterial pathogens.

ImmunoPET combines the sensitive PET imaging modality with the specificity of antibodies to image a biological target (116, 117). A prominent radiotracer is under development for *A. fumigatus* based on the humanized monoclonal antibody JF5 (hJF5mAb) radiolabeled with copper-64 (^64^Cu) named ([^64^Cu]Cu-NODAGA-hJF5 using immunoPET/MRI (118, 119). JF5mAb was found to bind to extracellular (galacto) mannoprotein antigens in clinically relevant *Aspergillus* species *via* lateral-flow assays. It was able to detect Aspergillus infection in the lung in an in vivo pulmonary aspergillosis infection model in neutropenic mice (119). Though Ab-mediated imaging diagnostics ought to ensure the specificity of the radiotracers, antibodies have an extended circulation time (>2 days), poor target penetration and significant off-target liver retention, and the cost of the radiotracer could be high due to the production of clinical grade monoclonal antibodies (120).

#### 3.2.3 Antimicrobial peptides and antibiotics

Various antimicrobial peptides (AMPs) have been investigated, and particularly human respiratory epithelium-specific cationic UBI fragments (29–41) labeled with ^99m^Tc ([^99m^Tc]Tc-UBI-29–41) allowed preclinical detection of *S. aureus* in a murine myositis model (29, 30). In a further clinical study with a small group of patients with suspected bone, soft-tissue, or prosthesis infections, the researchers were able to localize infectious foci, with optimal visualization at 30 min (30). This promising AMP was further labeled with ^68^Ga with NOTA and DOTA, mostly used BFCs, and was found to retain its infection detection capability in preclinical experiments (31). Patients with suspected infection associated with diabetic foot disease, cellulitis and fractures were selected for the pilot study investigation to evaluate the use of the tracer [^68^Ga]Ga-DOTA-UBI in localization of infection sites (121). Although preclinical results were promising, it failed to identify infection in four patients with culture positive infection.

Numerous preclinical studies have been done using radiolabeled antibiotics or antimicrobial compounds to target various essential cellular processes such as cell wall, protein, and nucleic acid synthesis, but none of them are in clinical use. Since bacteria-specific antibiotics are designed to kill bacteria at the lowest possible concentration, they may not be ideal candidates for imaging purposes due to the lack of appreciable signal amplification from the tracer accumulation by the bacteria. Notable examples of radiolabeled antibiotics are [^99m^Tc]Tc-ciprofloxacin (122, 123), [^124^I]I-Fialuridine ([^124^I]I-FIAU) for *S. aureus* (124) and [^99m^Tc]Tcamphotericin B ([^99m^Tc]Tc-AMB) (125).

The specificity and sensitivity for infections of [^99m^Tc]Tc-ciprofloxacin have been investigated in several studies, probably due to the formation of several radiolabeled chemical species with different biodistribution profiles (126, 127). Fialuridine is a nucleoside analogue that is a substrate for the bacterial thymidine kinase enzyme but not acted on by the human form of the enzyme, and forms the basis of a potential molecular probe for infection imaging. However, [^124^I]I-FIAU lacks specificity in patients with prosthetic joint infections, and it has a high background signal in uninfected muscle, presumably due to host mitochondrial metabolism (128). While disappointing results were seen with [^99m^Tc]Tc-ciprofloxacin and [^124^I]I-FIAU, the [^99m^Tc]Tc-AMB showed mixed results with uptake in *S. aureus* along with *A. fumigatus*. Another recent *in vitro* study revisited [^99m^Tc]Tc-AMB, and also AMB labeled with ^68^Ga, and identified uptake in *A. fumigatus* and *Rhizopus arrhizus* (129). Radiolabeled AMB was also investigated in a murine model of invasive mycoses with subcutaneous *C. albicans* and *A. niger* infection (125). In contrast, [^99m^Tc]Tc-fluconazole successfully detected *C. albicans* but not *A. fumigatus* infection (130). [^99m^Tc]Tc-tricarbonylcaspofungin was promising in scintigraphic imaging studies of *C. albicans* and *A. niger* infections in mice (131).

[^18^F]F-trimethoprim was evaluated to detect infection caused by *E. coli*, *P. aeruginosa* and *S. aureus* and showed specificity for muscle infection *in vivo* (132). Phase 1 clinical studies are actively recruiting patients to evaluate [^18^F]F-trimethoprim and [^11^C]C-trimethoprim as tracers for bacterial infection, registered at ClinicalTrials.gov as NCT03424525 (133) and NCT04263792 (134), respectively.]

Radiolabeled antimycobacterials, for example, 2-[^18^F]fluoroisoniazid were reported to accumulate in a tuberculosis lung infection model more rapidly than 18FDG (135), but there was no non-infected control group. A recent study has evaluated bromine-76 (^76^Br) radiolabeled bedaquiline to develop TB specific radiotracer (136). In a further pulmonary TB infection model in mice, [^76^Br]Br-bedaquiline PET imaging showed selective localization in adipose tissue, excellent penetration into infected lung lesions, and measurable off target uptake in the brain parenchyma.

**Figure 5:**
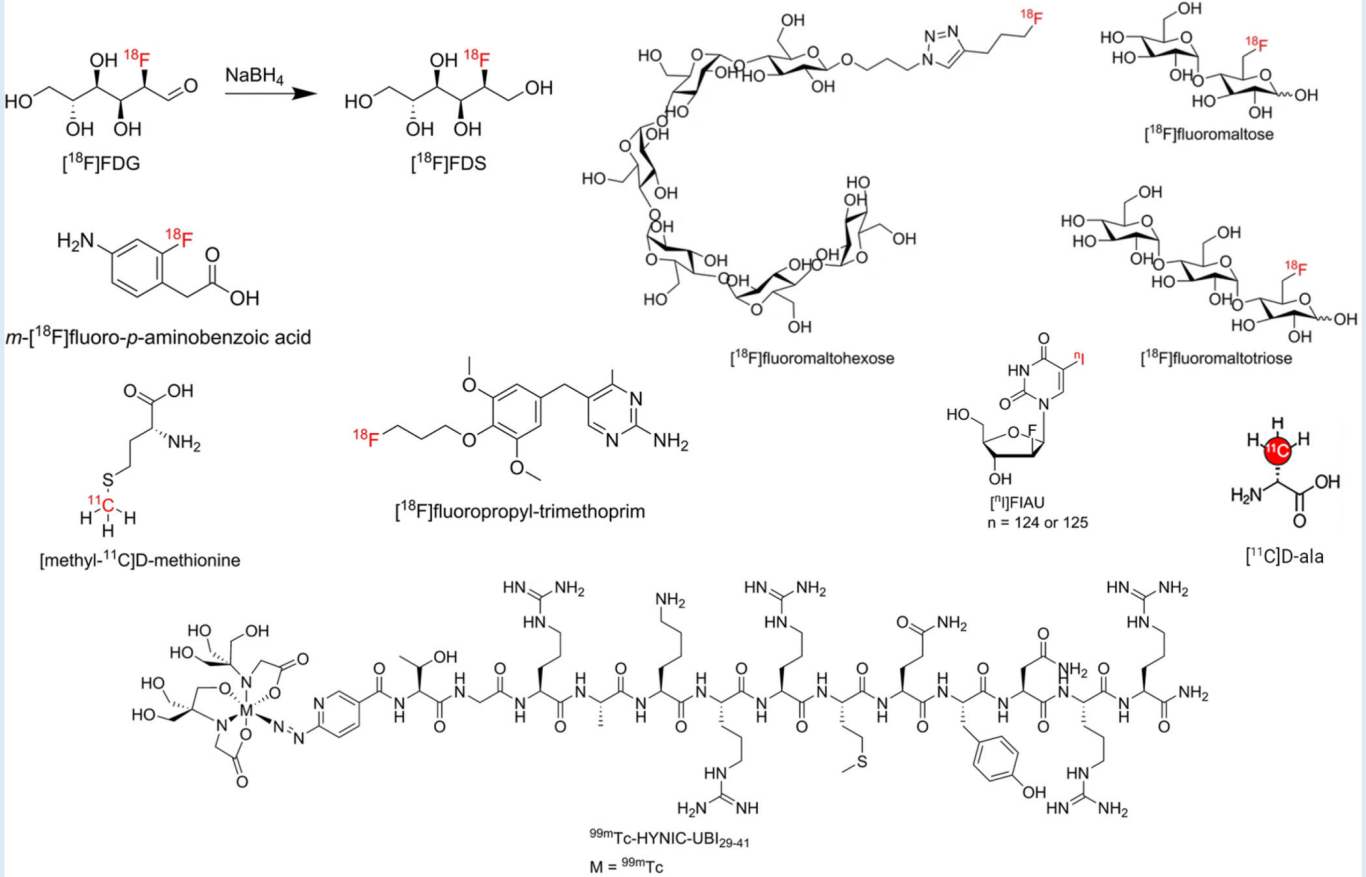
Structure of some radiotracers mentioned in Section 3.2.1–3.2.3. Reproduced from References (117, 137) with permission under Creative Commons Attribution 4.0 International Licence. Reproduced with BioRender.com.

Figure 5 has highlighted some of the structures of radiotracers mentioned in Section 3.2.1–3.2.3.

#### 3.2.4 Transition and trace metals ions

Transition and trace metals ions, including iron (Fe), zinc (Zn), manganese (Mn), and copper (Cu) are essential for many host and microbial cellular processes (138, 139). Transport of these metals is often distinct in bacteria and fungi (and humans) which could be exploited to design pathogen specific tracers. Metal acquisition is achieved by secreting metal-chelating molecules using dedicated membrane transport systems (140). Recent metal homeostasis studies have deciphered the function in copper homeostasis of two transport proteins, ferric yersiniabactin uptake A (FyuA) in *E. coli* and metal-staphylopine-binding protein (CntA) in *S. aureus* (141, 142). In *S. aureus* staphylopine is able to chelate a range of metals including nickel, cobalt, zinc, copper, and iron. The metal acquisition affinity for staphylopine is Cu2+> Ni2+> Co^2+^> Zn^2+^> Fe^2+^. Genes encoding staphylopine are also conserved in pathogens such as *Yersinia pestis* and *P. aeruginosa*. These findings provide the opportunity to explore transport pathways of metals, using nuclear medicine, to develop radiometal chelation-based microbial imaging diagnostics.

**Figure 6:**
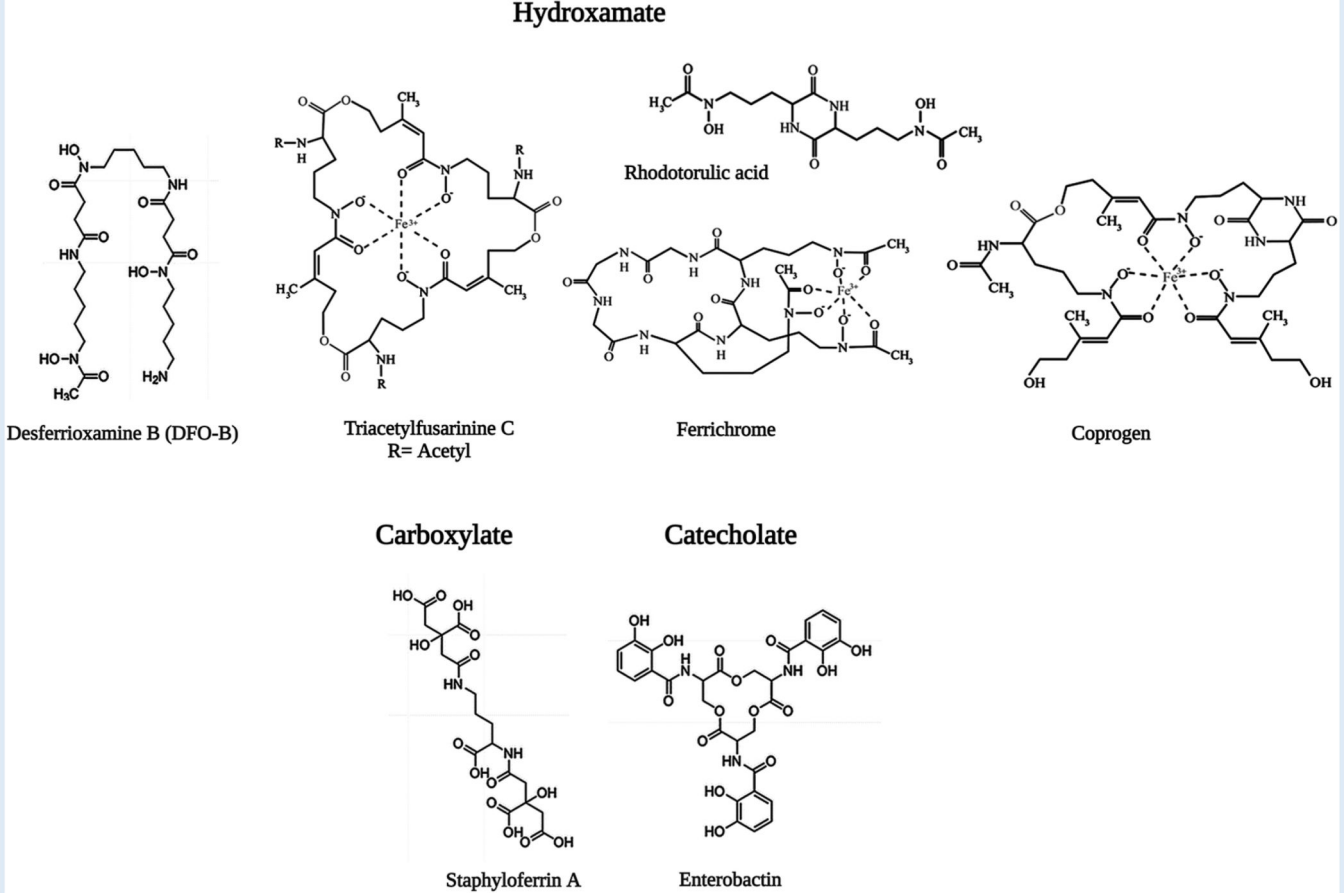
Representative structure of microbial siderophores. Triacetylfusarinine (TAFC), ferrichrome and coprogen are shown in the ferric form, In TAFC, R = acetyl. Reproduced from References (147, 148) with permission under Creative Commons Attribution 4.0 International Licence and Creative Commons Attribution 3.0 Unported Licence, respectively. Reproduced with BioRender.com.

**Figure 7:**
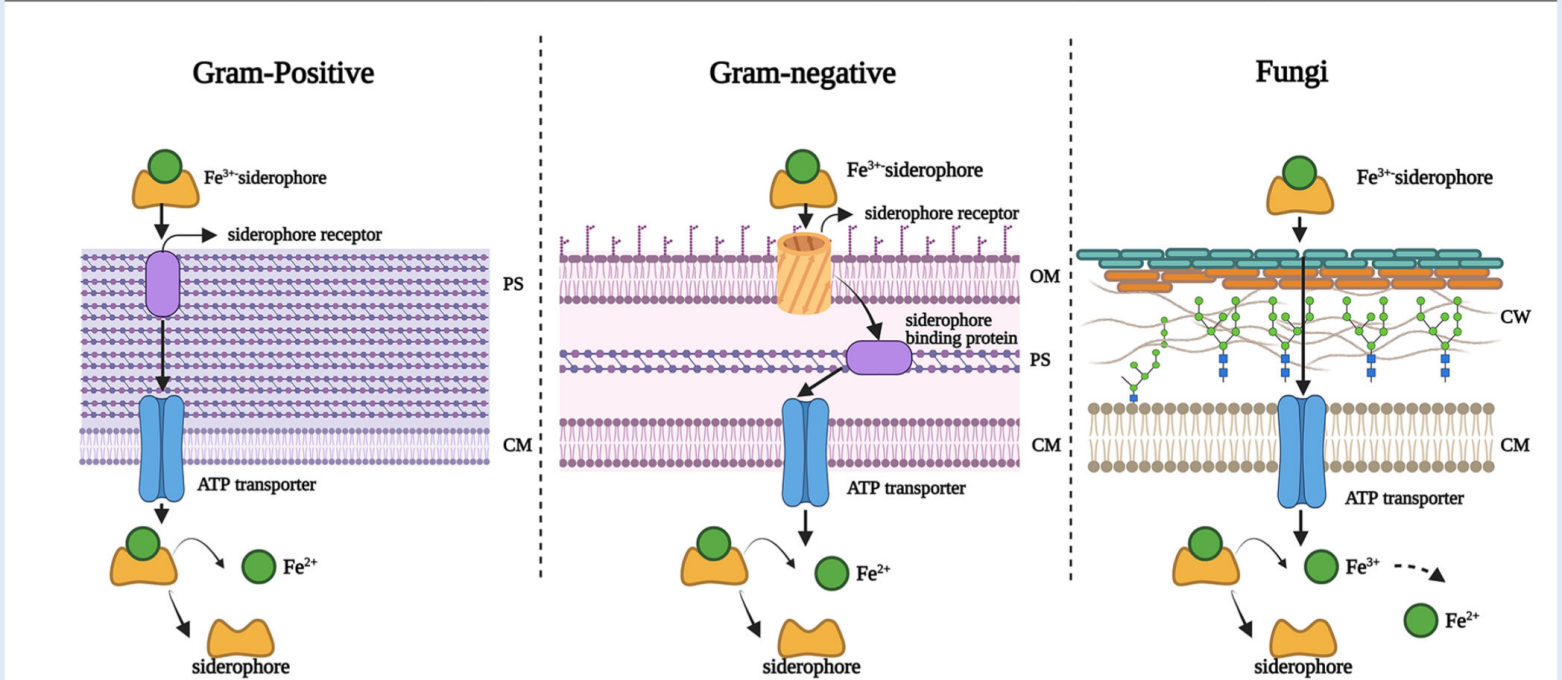
Mechanisms of siderophore-mediated iron transport in Gram-positive bacteria, Gram-negative bacteria and fungi. PS: periplasmic space; CM: cell membrane; OM: outer membrane; CW: cell wall. Created with BioRender.com.

Iron is the most essential nutrient among these metals and plays a key factor in the virulence of pathogenic bacteria and fungi (143). During infection, pathogens encounter an almost iron-free environment as the available iron is tightly sequestered by host proteins, e.g., hemoglobin, transferrin, lactoferrin, and ferritin. To overcome this situation, bacteria and fungi secrete small iron-binding siderophores molecules and express cell-surface active siderophore uptake receptors. Siderophores have high iron binding affinities and sequester Fe(III) from the environment, and microorganisms actively internalize it via these receptors (144). So far more than 500 different siderophores have been identified (145). They are chemically diverse and can be categorized into hydroxamate, catechol, carboxylate, phenolate and mixed types. These groups are involved in iron-complexing processes, with abilities to form tighter complexes according to their chemical geometry, and number of donors within the same molecule. The chemistry and biology of siderophores have been extensively reviewed elsewhere (146). Some representative structures of siderophores from bacteria and fungi are depicted in Figure 6. There are distinct mechanisms of siderophore-mediated iron transport in Gram-positive and Gram-negative bacteria and fungi (Figure 7). These iron acquisition systems are unique to microorganisms and plants; humans do not produce them.

Desferrioxamine B (DFO-B) is a clinically approved drug known as Desferal (Novartis), used to treat iron overload in patients, and produced by *Streptomyces pilosus* (149). Although siderophores are specific to different types of bacteria and fungi, many bacteria/fungi can utilize siderophores which are not their own (called xenosiderophores) (144). Interestingly some unicellular fungi, for instance *Saccharomyces cerevisiae* and *Candida* spp., do not produce siderophores but can internalize them *via* siderophore receptors in their cell membrane (150, 151). Table 4 summarizes the native siderophores and xenosiderophores in selected important pathogens.

As mentioned in Section 1, recent progress has been made with siderophore-based radiotracer development by utilizing the specificity of siderophores in microorganisms, therefore the following section will further discuss the potential of siderophore-based pathogen specific radiotracers development in more detail.

### 3.3 Recent development of siderophore based metal chelators

Radiolabeled siderophores have been investigated since the early 1980s (170). [^111^In]In-DFO-B has been proposed for abscess detection. Studies were carried out in rabbits with turpentine-induced abscess and *Staphylococcus*-induced abscess. Images at 4 h and 24 h show the abscess clearly visible (171).

Over the years, gallium compounds have been particularly important in the development of radiopharmaceuticals (172). The chemical and biological similarities between Gallium(III) (Ga^3+^) and Iron(III) (Fe^3+^) are key to the use of Ga^3+^ (radioisotopes) in many applications (173). Non-radioactive gallium compounds were also investigated for cancer diagnostics (174). The similarity between Ga^3+^ to Fe^3+^ enables radioactive gallium (^68/67^Ga) bound to siderophores as BFCs for investigating many biological applications (Supplementary Table S1).

For infection imaging purposes, a ^67^Ga-labeled analogue of ferrichrome has shown *in vitro* uptake in the fungus *Ustilago sphaerogena via* an active mechanism that was indistinguishable from non-radiolabeled ferrichrome (175). Recent preclinical studies have been exploring a range of siderophores to develop ^68^Ga-labeled-siderophore based PET tracers for bacterial and fungal infection.

*In vitro* evaluation of siderophores mainly targeting *A. fumigatus* has been conducted (176). [^68^Ga]Ga-desferri-ferricrocin ([^68^Ga]Ga-FC) and [^68^Ga]Ga-desferri-triacetylfusarinine C ([^68^Ga]Ga-TAFC) were evaluated *in vitro*, and *in vivo* in a rat lung infection model. Though both siderophores bound 68Ga with high affinity, [^68^Ga]Ga-TAFC showed high stability. Furthermore, [^68^Ga]Ga-TAFC exhibited infection site (lung) accumulation *in vivo*. Later, the same group included [^68^Ga]Ga-TAFC, [^68^Ga]Ga-ferrioxamine E (FOXE), [^68^Ga]Ga-FC, [^68^Ga]Ga-ferrichrome and [^68^Ga]Ga-fusarinine C to evaluate *in vitro* and pharmacokinetics (PK) in an *in vivo* healthy murine model (177). They confirmed the suitability of [^68^Ga]Ga-TAFC and [^68^Ga]Ga-FOXE for use in further animal infection models. They then used [^68^Ga]Ga-TAFC and [^68^Ga]Ga-FOXE in *in vivo* neutropenic rats with pulmonary aspergillosis and used PET/CT to demonstrate their ability to detect infection (178). They also confirmed no uptake of these two potential tracers in human lung tissue by *ex vivo* uptake assay. However, this study did not consider iron-overload conditions in neutropenic patients in their infection model study, and therefore the effect of not pretreating with iron chelators to reduce free iron (to stop transchelation with TAFC and FOXE) was not evaluated. This could be the reason for the slightly reduced uptake observed in the rat model compared to high *in vitro* uptake results.

After initial promising results, the same group examined the *in vitro* specificity of [^68^Ga]Ga-TAFC and [^68^Ga]Ga-FOXE in other microorganisms (bacteria, fungi and yeast) and showed rapid and higher uptake in *A. fumigatus*, lower uptake in other fungi, and nearly no uptake in other bacteria and yeasts (179). However, *in vitro* specificity assays in various microorganisms should ideally have been performed before any *in vivo A. fumigatus* infection model in order to confirm the specificity of the tracer. *In vitro* results after 90 min showed uptake in *A. flavus*, *A. terreus*, and lower uptake in *R. oryzae* and *Fusarium solani* compared to *A. fumigatus* for both tracers, mainly in iron-deficient conditions. [^68^Ga]Ga-FOXE was also shown to be taken up by *S. aureus in vitro*. In contrast, the abscess model of *S. aureus* did not show any uptake *in vivo*. Furthermore, there was noticeable uptake of [^68^Ga]Ga-TAFC and [^68^Ga]Ga-FOXE in the area of induced sterile inflammation. The authors suggested the severe induced inflammation was the reason for this non-specific uptake of both tracers.

Later they also compared the *in vivo* PK of [^68^Ga]Ga-TAFC and [^68^Ga]Ga-FOXE, ^68^Ga-colloids and [^68^Ga]Ga-citrate in a healthy murine model (180). This showed the same rapid renal excretion and low blood retention of [^68^Ga]Ga-TAFC and [^68^Ga]Ga-FOXE. Some gastrointestinal retention was observed for [^68^Ga]Ga-FOXE, but overall, the results were comparable with colloid and citrate.

Although Petrik et al. showed preclinical progress with [^68^Ga]Ga-TAFC and [^68^Ga]Ga-FOXE, these tracers have not progressed to early phase clinical studies. Instead, they evaluated *P. aeruginosa* specific siderophore screening for infection diagnostics (181). *P. aeruginosa* produces different types of siderophores including pyoverdine (PVD) and uses highly efficient FpvA transporters specific for ferripyoverdine uptake. PVD is involved in nutrition, biofilm control, cell-to-cell communication and virulence regulation. [^68^Ga]Ga-PVD-PAO1 (named after the strain name) showed excellent PK properties in healthy mice with rapid renal elimination. Further study in a murine acute respiratory and muscle infection model demonstrated selective accumulation of the radiotracer in infected tissues. The study also demonstrated great sensitivity by enabling the detection of only five viable cells of *P. aeruginosa in vivo*. Although from published data, aerobactin would be expected to be taken up by *P. aeruginosa*, their in vitro results showed very low uptake of aerobactin. The in vitro specificity of [^68^Ga]GaPVD-PO1 was good in *P. aeruginosa* strains (almost no uptake in other tested bacteria and *C. albicans*). This promising result warrants further clinical studies in human lung infections.

Another study was conducted to develop ^67^Ga-labeled PET tracer with DFO-B for its potential smooth translation to clinical practice to image *S. aureus* infection (182). Gram-positive *S. aureus* infection was targeted for *in vitro* and *in vivo* uptake of [^67^Ga]Ga-DFO-B, though the authors concluded natural DFO-B is not an ideal candidate to act as an infection imaging agent in patients due its very fast clearance in an in vivo animal infection model. Instead, they developed DFO-derivatives with improved PK. The main aim was to improve the pharmacokinetics of DFO-derived radiotracers with increased uptake in bacteria (*S. aureus*). When compared with the control [^67^Ga]Ga-16, none of the derivatives shows high *in vitro* uptake in *S. aureus*. Additionally, three potential derivatives [^67^Ga]Ga-18, [^67^Ga]Ga-26, and [^67^Ga]Ga-28 were compared with the control in a mouse myositis infection model. Results showed 6 : 1 and 11 : 1 infected and non-infected tissue ratios in control and [^67^Ga]Ga-18, respectively. For the remaining two tracers, the ratio was minimal. However, they did not perform PET imaging to support their findings. Moreover, the ex vivo biodistribution showed high uptake in the small intestine, liver, and gallbladder for all three derivatives. Therefore, these derivatives did not achieve their set goals since the *in vitro* and *in vivo* uptake in *S. aureus* was not greatly improved in these derivatives.

**Figure 8:**
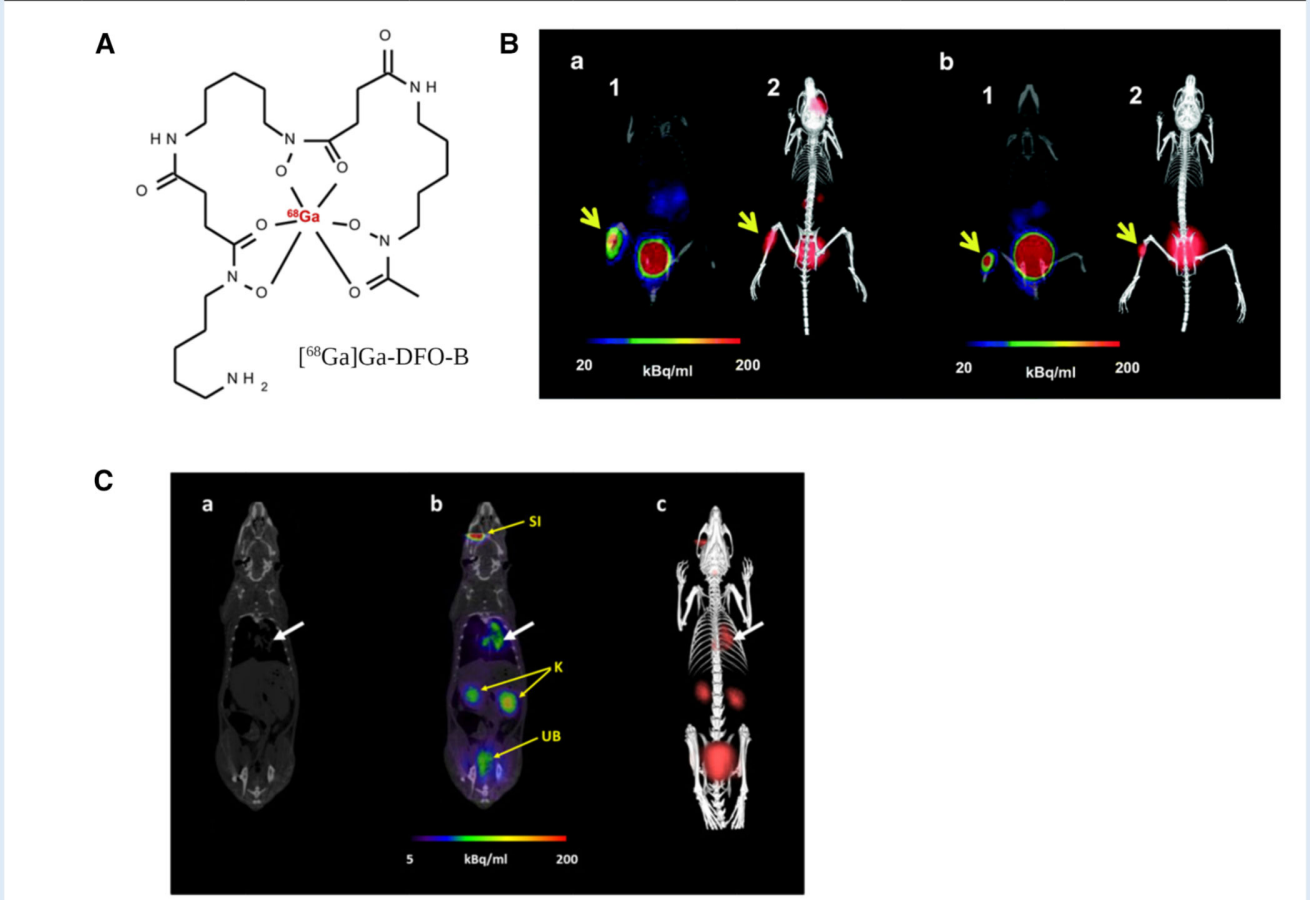
PET/CT imaging with [^68^Ga]Ga-DFO-B-in vivo mice infection model. (**A**) structure of [^68^Ga]Ga-DFO-B. (**B**) Static PET/CT imaging (coronal slices (1) and 3D volume rendered images (2)) of [^68^Ga]Ga-DFO-B in *P. aeruginosa* infected (**a**) and *S. aureus* infected (**b**) Balb/c mice 45 min after injection. (C) Static PET/CT images of [^68^Ga]Ga-DFO-B in pulmonary *A. fumigatus*-infected Lewis rats 45 min after injection: (**a**) CT coronal slice, (**b**) fused PET/CT coronal slice and (**c**) 3D volume rendered PET/CT image. White arrow indicates *A. fumigatus* infection, SI = site of injection, K = kidneys, UB = urinary bladder. Reproduced from References (183, 184) with permission under Creative Commons Attribution 4.0 International Licence. Reproduced with BioRender.com.

A recent study with [^68^Ga]Ga-DFO-B (Figure 8A) without any chemical modification has shown the potential of a DFO-Bbased PET tracer to image infections *in vitro* caused by *P. aeruginosa*, *S. aureus* and *S. agalactiae* (183) (Figure 8B). They used an *in vivo* infection model and PET/CT imaging showing infection imaging ability of [^68^Ga]Ga-DFO-B for these bacterial strains. No *in vitro* uptake was seen for *E. coli* and *C. albicans*. More recently, the same group repeated *in vitro* uptake of [^68^Ga]Ga-DFO-B in *A. fumigatus* and showed the uptake is pH dependent. They supported their finding with *in vivo* PET/CT imaging of pulmonary *A. fumigatus* infection in a rat model (184) (Figure 8C). This finding is contrary to their previous *in vitro* uptake results, where *in vivo* infection model was not included. Although this group has achieved repurposing of DFO-B as a broad-spectrum infection imaging tracer experimentally, it necessitates further human studies in improved disease models.

Introduction of fluorescent dye (185) to produce a hybrid imaging modality resulted in rapid uptake in *A. fumigatus in vitro*, and in an infected lung model in rats showed high fluorescent signal levels that were comparable to a PET/CT scan. This demonstrates the potential applicability of siderophores for hybrid imaging, with possible applications in surgical procedures and bioluminescence in humans (186). A group of researchers in the UK did some preliminary *in vitro* uptake of [^68^Ga]Ga-ferrichrome C (a fungal siderophore) using *E. coli* in iron-deficient medium, and in vascular stents incubated with *E. coli* cells (187). Interestingly, this study performed PET/CT imaging of stents incubated with *E. coli* vs. sterile stents which shows the applicability of this technique for stent or other medical implant-associated infections. However, there were no details about the methodology in their publication. The same group is now launching an observational clinical study (Clinicaltrials.gov NCT05285072) with [^68^Ga]Ga-DFO-B for VGI imaging titled as “Siderophores for Imaging Infection: A first-in-human pilot study using a tracer ([^68^Ga]Ga-DFO-B) as proof of concept that siderophores can image bacterial infection in vascular grafts” (188). Table 5 summarizes current *in vitro* and *in vivo* investigations of ^67/68^Ga-siderophores for infection imaging.

Cyclotron produced radiometal Zirconium-89 (^89^Zr) is promising for clinical immune-PET applications, mainly due to its longer half-life that matches the biological half-life of antibodies, and by being safe for human use by virtue of its biological inertness (80). Though the chemistry of ^89^Zr is not very close to Iron, it does behave somewhat similarly to Iron thus it can form a stable radiocomplex with siderophores (190). For infection diagnostics, [^89^Zr]Zr-DFO-B was conjugated with gram-positive specific SAC55, a mAb raised against lipoteichoic acid (LTA), and was used to image an inoculated femur implant in a murine model. When compared to 18FDG and other controls it showed potential differentiation between infection and sterile inflammation (191). Siderophores TAFC and FOXE, selected for *A. fumigatus*, were also evaluated by labelling with ^89^Zr and compared with [^68^Ga]Ga-TAFC and [^68^Ga]Ga-FOXE to assess the possibility of siderophore-bioconjugates (antibodies/peptides) with ^89^Zr (192). The results showed parallel in vitro and in vivo characteristics with ^68^Ga and ^89^Zr, except [^89^Zr]Zr-FOXE. Another study evaluated the infection imaging potential of ^89^Zr chelators in detecting both *S. aureus* and *P. aeruginosa* (193). In an *in vivo* mouse lung infection model using *P. aeruginosa* there was no significant differences between infection and control mice as determined by biodistribution.

Another cyclotron produced radiometal, ^64^Cu is of interest as siderophores can chelate metals other than iron. ^64^Cu has been conjugated with the siderophore yersiniabactin (YbT) and in *in vitro* studies with *E. coli* it exhibited uptake of [^64^Cu]Cu-YbT (189). This uptake is regulated by the FyuA transporter protein present in *E. coli*. *In vivo* studies with *E. coli* strains UTI and Nissle, *K. pneumoniae*, *P. aeruginosa*, and *S. aureus* in a murine muscle model has demonstrated the uptake of the tracers by all strains except *S. aureus* (because *S. aureus* does not possess FyuA transporter protein) (141, 194).

### 3.4 Other progress in radionuclide-based/optical-based infection imaging

[^68^Ga]Ga-DOTA-TOC, is a PET tracer used for the diagnosis and follow-up of neuroendocrine tumors (NETs) (195). Though this tracer has a high binding affinity to somatostatin receptor subtype 2 (SSTR2), it also binds to somatostatin receptor subtype 5 (SSTR5). Since somatostatin receptors are expressed by activated monocytes, macrophages, and lymphocytes, detection of SSTR2 receptors could potentially be of utility in the diagnosis of IE and other infections. A phase 2 clinical trial (NCT05183555) is underway (196).

A collaborative clinical study with 18FDG PET/CT in *S. aureus* Bacteremia (PET-SAB) has been launched recently in the UK, registered as NCT05361135 (197). The study aims to include 520 patients and terminate in 2026.

Targeted optical and fluorescence imaging can localize pathogen specific prothrombin activation during *S. aureus* endocarditis by targeting blood coagulation phenomena (198). This was capable of detecting blood coagulation by *S. aureus in vivo*, but was unable to visualize the actual bacteria at the site of infection. [^68^Ga]Ga-apo-transferrin also showed capability of *S. aureus* infection in a murine myositis model (199).

Vancomycin labeled with a fluorophore, IRDye 800CW, was used to detect *S. aureus* in an *in vivo* myositis model by injecting engineered luciferase expressing strain of *S. aureus*. The study demonstrated the potential of fluorophore-labeled vancomycin to detect *S. aureus* infection with a high signal to noise ratio. However, this strategy would only detect Gram-positive bacteria and would not be able to differentiate between different species of Gram-positive bacteria (200). Fluorescence-PET for *S. aureus* detection with a human mAb probe has also been investigated (201, 202). None of these are in clinical use yet, as these optical imaging using fluorescence-PET tracers are currently only applicable to visualization of superficial infections due to their limited tissue penetration.

## 4 Challenges to development of siderophore-based infection diagnostics

Development of pathogen-specific tracers has been challenging and has demonstrated variable results. Recently, ^68^Ga use in particular is showing dramatic progress because of its applicability in labeling of diverse range of compounds coupled with smooth clinical translation and low radiation burden to patients. Though ^68^Ga-based PET radiotracers targeting infectious agents are currently in progress, only well-investigated tracers with low immunogenicity and toxicity will have a high likelihood of clinical introduction. Such diagnostic tracers should have high sensitivity and high positive and negative predictive values, clear distinction of inflammation vs. microbial infection, specificity towards desired bacterial or fungal pathogens and feasibility of low-cost production. Multimodal imaging capability and theranostic applications would be advantageous. The tracer should target both metabolically active and quiescent pathogens within biofilm, as well as antibiotic susceptible (unable to grow in the presence of antibiotic) and resistant pathogens. Pathogen-specific imaging tracers could help in establishing species-specific diagnoses of bacterial infections that are not amenable to detection by traditional tools, monitor and prognosticate treatment effects, and rationalize antimicrobial use. ^68^Ga-siderophores are currently showing promise to develop such radiotracers for microbial specific noninvasive whole-body PET/CT imaging diagnostics. Moreover, the currently planned clinical study in VGI patients with [^68^Ga]Ga-DFO-B will lead the way for clinical translation of the ^68^Ga-siderophore approach.

Though research with other siderophores is promising, there are several challenges to address before this approach becomes regular clinical practice.

### 4.1 Microbial challenges

#### 4.1.1 Imaging biofilms and persister cells

Biofilms are coordinated associations of microbial cells that adhere to surfaces by producing an extracellular matrix (ECM). This ECM consists of exopolysaccharides (EPS), extracellular DNA (eDNA) and various proteins, and are characteristic amongst different species (203). Biofilms can be associated with a medically implanted device (such as vascular grafts, prosthetics, catheters, etc.) or non-device related infections (such as endocarditis, lung infection in CF, urinary tract, osteomyelitis etc.). These infections involving biofilm account for more than two thirds of the chronic and recurrent infections in humans and increase the risk of AMR globally due to their inherent ability to resist antibiotic penetration (204). For example, in VGI, both *S. aureus* (Supplementary Figure S1) and CoNS possess virulence factors that facilitate their adherence to prosthetic materials. Oxacillin-resistant *S. aureus* and *P. aeruginosa* infections are usually associated with more severe infections and may result in higher rates of morbidity and mortality compared with low virulence organisms such as CoNS, *Corynebacterium*, or *Propionibacterium* species. Infections due to Gram-negative bacteria such as *E. coli*, *Pseudomonas*, *Klebsiella*, *Enterobacter* and *Proteus* spp. may also be virulent.

Although biofilm infections are most commonly chronic and localized, prolonged presence of biofilms may result in occult or asymptomatic infections that are only detectable when they cause bacteremia (205). Moreover, some infections harbor polymicrobial biofilms, which in turn are very difficult to diagnose and treat. For example, CF patients may develop lung infections with polymicrobial biofilm infection including a combination of bacteria and fungi.

Antimicrobial therapy may not be effective for several reasons, for example if pathogens are not susceptible due to the slow rate of replication rendering common antimicrobial therapeutic targets ineffective. The drugs may not reach the site of the infection or not in adequate concentrations. Persister cells are the slow growing normal cells that remain dormant, and randomly develop antimicrobial tolerance by genetic mutation. For example, in CF patients *P. aeruginosa* persister cells were identified (206). In patients with oral thrush biofilms, persister *C. albicans* can also be found (207). When biofilm is present in an implanted device, bacteria may not be detected until the device is surgically removed from the patient and samples are cultured. However, false negative results may occur if slow-growing bacteria and persister cells are present and they do not form colonies under routine culture techniques (208, 209). Culture and detection of causative microbes may become problematic due to the heterogeneous distribution of biofilms and requirement of specific growth factors for mixed species (210). Sonication of samples may be beneficial. Though there is a guideline for diagnosis and treatment of biofilm infection (211), there is no commonly used clinical laboratory technique for direct detection of biofilm in explanted devices.

Current imaging techniques that detect the host response to infection do not visualize biofilm and cannot determine whether live organisms remain within biofilm or the surrounding tissues. 18FDG PET/CT has great utility in the diagnosis of vascular graft infection but remains non-specific. It is also not able to accurately determine the efficacy of treatment or whether an adequate duration of treatment has been administered due to persisting host sterile inflammatory metabolic activity after the infective organism has been killed. This makes determining the length of an antimicrobial therapeutic course very difficult, and most patients require major surgery or get treated with lifelong antimicrobial therapy. This is no high-quality evidence to guide the management of vascular prosthetic infection (212, 213). Unfortunately, no significant investigation has been published on direct imaging of biofilm infection *in vivo*. Research is being conducted to detect biofilm preclinically either by developing human monoclonal Abs against *S. aureus* surface antigens (214) or exploring receptor of quorum sensing (QS) molecules (215), anti-biofilm antibodies (216), and peptide-based probes targeting bacterial-specific biofilms (217). This may form the next challenge for image-based diagnostics.

#### 4.1.2 Microbial cell numbers in the site of infection

To develop a diagnostic tool for a particular infection, microbial cell number is crucial as the number of infectious cells may vary depending on the site of the infection, type of pathogens and host immunity. Early diagnosis of any infection means acute infection with low to high number of pathogens depending on the infection site and nature of the pathogen and the immunity of the individual. For instance, a preclinical study with graft infection with *P. aeruginosa* showed more than half of the canine models with aortic grafts had as low as 10^2^ cells (218). Whereas ventilator-associated pneumonia (VAP) has a range of quantitative cultures, from 10^3^ to 10^6^ CFU/mL (219). Higher bacterial number as high as 10^9^ CFU/mL in sputum may be found with persistent lung infection (220). However, if an immunocompromised individual is considered, they may develop infection with a low number of pathogens. Additionally, if chronic infection is considered, sometimes biofilm leads to the failure of antimicrobial therapy. Therefore, it is important to consider the type of infections and the status of infection when developing diagnostics for them, and early diagnosis is preferrable for the infections where non-specific clinical symptoms are presented, and for high-risk immunocompromised individuals.

#### 4.1.3 Differentiation between commensal microorganisms

In addition to mixed pathogens (or polymicrobial biofilms), the presence of commensal microflora which are the normal colonizers of the human body might hinder the specific detection of pathogenic bacteria in the site of infection. In general, pathogenic microbes possess virulence factors for invasion, and commensal microbes either do not possess or express any virulence factors, and thus may not stimulate the host immune response system (220). Therefore, host immune system/factors are important to react against any components of virulent microorganisms. Another consideration of opportunistic pathogens where commensal microflora become pathogenic, for example, in immunocompromised patients. Moreover, commensal microflora become pathogens depending on the anatomical sites and local microenvironment (221). For instance, skin and mucous membrane commensal *S. aureus* can become invasive *via* breaches in skin or mucous membranes.

It is known that siderophores have an important role in virulence for pathogens and their production is upregulated during infection. Therefore, it is plausible that siderophore-based radiotracers could localize infection caused due to pathogenic bacteria rather than commensal bacteria, by multiplying the signal in PET scan. This hypothesis will be informed by the results from the observational clinical study of infection imaging with [^68^Ga]Ga-DFO-B.

### 4.2 Experimental challenges

#### 4.2.1 Lack of standardized preclinical research

Standardized preclinical research models are crucial to development of infection-specific tracers. Radiosynthesis of chemically stable radiotracers is the initial step, followed by *in vitro* screening in microorganisms and *in vivo* PK of the radiotracers (222). There is no doubt that depending on the targeted infections and/or targeted patients, large scale *in vitro* screening in different microorganisms, from both laboratory and clinical strains, should be performed. The limiting step could be the unfavorable PK of the potential radiotracers in healthy animal models. At this stage, chemical modification of the radiotracer is sought which will delay the further progress of the preclinical studies. If the potential tracers from *in vitro* studies show favorable PK like *in vivo* chemical stability, ability to accumulate in the site but at the same time low uptake in major organs, and relatively fast clearance from the body, *in vivo* infection model studies should follow. The outcome of these studies depends on the choice of appropriate infection model matched to the PK of the potential radiotracer. For instance, lipophilic radiotracers tend to penetrate membranes inside the body due to their ability to integrate into lipophilic membranes, and have slower clearance. This type of radiotracer is usually non-specific about the location of an infection and tends to accumulate in the liver. On the other hand, hydrophilic radiotracers tend to be less absorbed by any major organs, and usually show fast clearance from the body *via* the kidneys. Such radiotracers require a specific membrane transport system to enter the microbial cells.

Radiosynthesis and quality control of a new radiotracer requires confirmation of radiolabeling, radiochemical purity (≥95%), stability of the complex in normal saline and human serum, lipophilicity or hydrophilicity, ability to bind to serum proteins, binding affinity to target, binding to eukaryotic cells, and trans-binding ability in the presence of other molecules or chelators. However, there are no standardized protocols or consensus guidelines for carrying out such *in vitro* studies for developing new radiotracers for infection imaging. Thus, researchers publishing results across different labs using their own protocols and instrumentation may be irreproducible and/or incomparable.

*In vitro* uptake studies of a stable radiotracer also suffer from lack of standardized guidelines (microbial strains and their growth condition, minimum and maximum microbial load as CFU/mL, uptake procedure, determination of uptake in microbial cells, use of appropriate control, competitive assays, live vs. dead microbial cells, temperature dependent assay, and so on). Though there are publications for *in vitro* uptake of ^68^Ga-siderophores (and other radiotracers) in bacteria and fungi by some research groups, studies are typically incomparable due to the use of individual protocols and experiment conditions.

*In vivo* infection model studies are the biggest challenge in preclinical research, since the proper choice of infection model is necessary to produce meaningful data that can be successfully translated into human clinical use. Again, there are no guidelines for the conduct or reporting of these studies, beyond the generic ARRIVE guidelines for reporting *in vivo* studies in general (223).

Standardized protocols and consensus guidelines are needed, including using various pathogens with their detailed specification, defined animal infection models, injection sites, microbial CFU/mL per injection, waiting time before PET/CT scan, image acquisition and interpretation of parameters, sacrificing time, CFU obtained after sacrifice, use of appropriate control, comparison with current tracers, etc., (in addition to standard reporting items in the ARRIVE guidelines, such a sex and age).

The most common infection model in use is the murine muscle infection model, as it is easy to perform and can give a relatively rapid insight of the ability of a new radiotracer to detect infection. However, it might not be the best model for clinically relevant native tissue or prosthetic infection, where tracers should travel to a specific location, interact with host factors, and ideally penetrate biofilms.

A Teflon cage model has been reported as a standardized reproducible model to study bacterial infection in small animals (224, 225). A Teflon cage is implanted under the skin through a small incision (5 mm) on the back of the mouse. This model could allow local injection of a defined bacterial CFU/bacterial mass, easy recovery of bacteria and count from the foreign body, radiotracer concentration-based study, and biofilm studies. To understand the effect of host factors, it is necessary to establish targeted infection with the causative pathogens, for example, dual models combining infection with, for example, a model of cancer or immunosuppression. However, big animal models like rabbits or pigs may be required to introduce vascular grafts in smaller vessels (226). This is often not practical due to their higher costs, space requirements, absence of animal model facilities, and lack of inbred and genetically modified strains. Nevertheless, the use of small-animal models for *in vivo* study with standardized protocols could improve the reproducibility of data.

#### 4.2.2 Questions to answer in preclinical studies

Although ^68^Ga-siderophores hold prospects of translation into human practice, there is still much needed not only to confirm the infection imaging capability, but also to answer some other experimental questions and may be relevant to other promising radiotracers, for example:

- Which timepoint of microbial infection is likely to give highest signal to noise ratio for infection imaging?
- Can ^68^Ga-siderophore-based radiotracers penetrate tissue necrosis or biofilm at infection sites?
- Can the radiotracer detect infection with low microbial load (CFU/mL)?
- Can chemical modification or artificial siderophores increase the sensitivity and/or specificity to targeted infection?
- Can these tracers be used for occult infections, and/or distinguish between commensal growth and pathogenic growth?

#### 4.2.3 Limited clinical study

Although there is no overall standardized protocol and consensus guidelines for developing radiotracers for microbial infection, preclinical studies have been conducted to image infection specially with radiolabeled UBI 29–41(227) which showed specific muscular uptake of the tracers compared to inflammation in osteomyelitis patients (228). To date, no human study with ^68^Ga-siderophore-based tracers have been performed, except one recruiting for investigating the biodistribution and human dosimetry, as an observational study in patients with VGI using [^68^Ga]Ga-DFO-B. Nevertheless, the ability to detect infection by [^68^Ga]Ga-DFO-B PET/CT imaging would require further human studies targeting different infections as a broad-spectrum imaging agent. Whilst comparison with current 18FDG used in clinical practice will be of utility, this cannot be used to assess the sensitivity and specificity of novel tracers, due to the imperfect performance of 18FDG PET/CT in current clinical use. This clinically approved siderophore could lead us to understand the basic questions of further improving the PK and pharmacodynamics (PD) of the siderophore-based tracers. Therefore, following questions are important to answer:

- Are the new tracers non-toxic and safe to administer to humans?
- What is the risk of allergic reaction?
- How will the optimal radiation dose be calculated for human administration?
- What is the minimum radiation dose for humans that could detect and locate infection without radiation burden to human subjects?
- Could simple kit-based radiotracers be developed for ease of clinical application?

#### 4.2.4 Regulation of clinical translation

Since DFO-B is already in clinical use, DFO-B radiolabeled with gallium-67/68 has less regulatory barriers to clinical translation and could be achieved in near future. Any potential new radiotracer should undergo its radiosynthesis *via* Good Manufacturing Practice (GMP) and the process through automated radiosynthesis set up to reduce the exposures to the radiochemist and to accelerate the overall process (229). Tracers other than [^68^Ga]Ga-DFO-B, must meet requirements set up by the relevant regulatory bodies (230, 231). Nevertheless, the regulatory requirements should be revised and set for novel radiopharmaceuticals in medicinal potential to overcome the barriers to their clinical translation (232, 233).

#### 4.2.5 Costs of PET/CT scanning

The drawbacks of PET radiotracers include the requirement for expensive equipment in the PET Centre, relatively high costs per scan (234), and inconsistent global availability of PET facilities. Hopefully, with the reduced costs of clinical grade gallium-68 generators, the automation of the radiosynthesis and quality control of the radiation dose preparation, costs may eventually reduce. Careful consideration of patient selection will allow use of this infection-specific imaging technique *via* ^68^Ga-siderophores and other radiotracers, which could reduce the total cost of PET/CT scan per year.

## 5 Future directions

For clinical practice, both broad and narrow spectrum imaging tracers are desirable (Supplementary Figure S2). Broad spectrum radiotracers could be useful when clinical manifestations are subtle, for example, FUO or detection of any infection in high-risk immunocompromised patients. Narrow spectrum radiotracers, on the other hand, could facilitate the detection and anatomical localization of specific bacterial or fungal infection in patients where pathological manifestation is already established. For example, VGI is often associated with Gram-positive *S. aureus* and CoNS (235), thus a radiotracer specifically detecting *S. aureus* would be useful for early detection and localization of the pathogen, if *S. aureus* was detected in blood cultures.

Among the most prominent imaging candidates are ^68^Ga labeled siderophores (Supplementary Table S2). Low molecular weight siderophore-based radiotracers (bound to an iron-memetic gallium radioisotope) have shown a hydrophilic nature and fast PK *in vivo* (with rapid clearance through urine). Since it plays an important role in essential iron transfer in microbial cells through a specific receptor system, it could fulfill the characteristics of being infection imaging radiotracers that can be transported to pathogenic bacteria and localize them specifically.

However, modification of existing siderophores (236, 237) or synthetic chelators with better specificity and better PK/PD properties would address their currently reduced specificity or non-applicability in certain disease models (e.g., ^68^Ga-siderophores are largely excreted *via* renal system, meaning invasive candidiasis affecting the kidney and ureteric tract cannot be detected with this approach). ^89^Zr-siderophores and ^64^Cusiderophores are also emerging and more research should be carried out to improve the chelating properties of siderophores and to biconjugate a range of specific antibodies (194, 238).

Siderochelin proteins in the human body can prevent iron acquisition in microorganisms by binding to siderophores, e.g., enterobactin (239). Therefore, choice of “stealth siderophores” that can evade human siderochelin binding mechanisms (e. g. salmochelin, yersiniabactin, pyoverdine) and/or chemical modification of existing potential siderophores could be evaluated.

**Figure 9:**
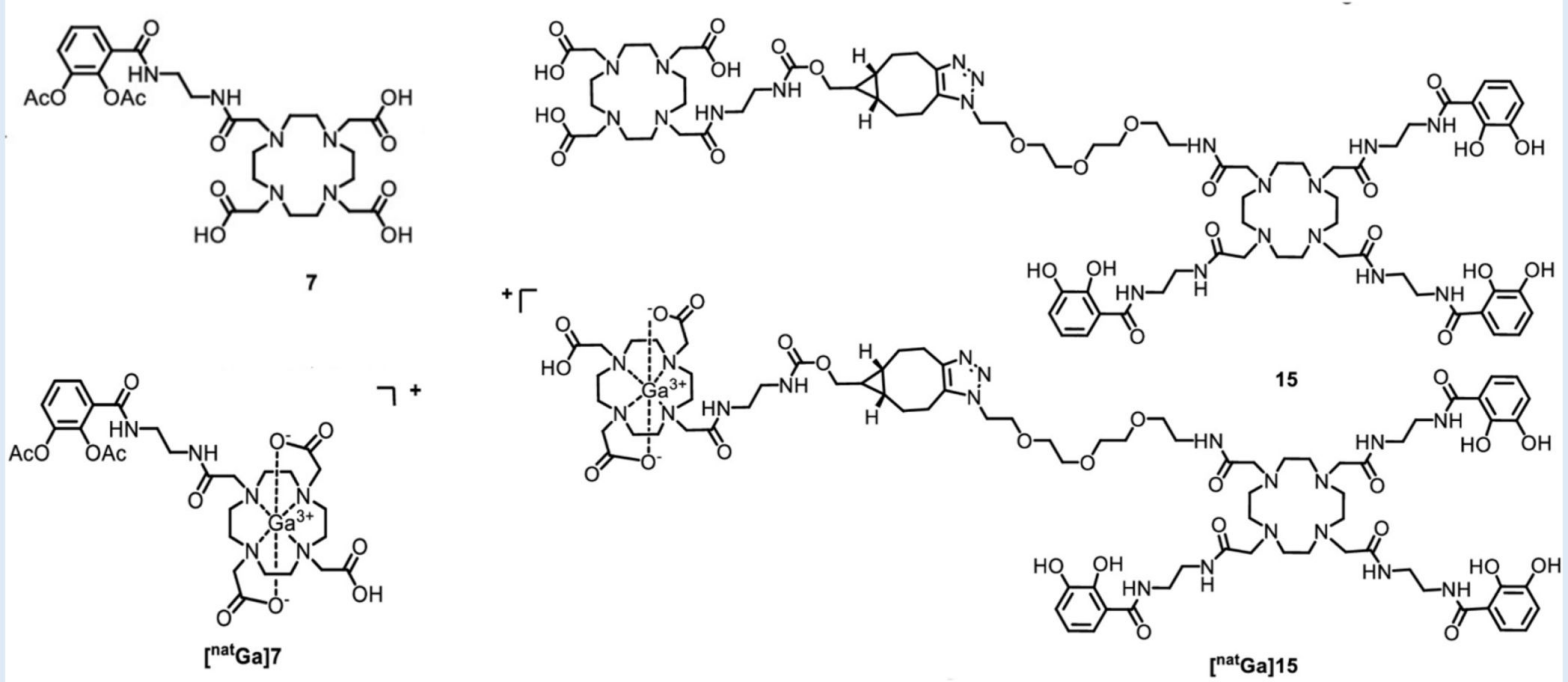
Structure of two artificial siderophores (7 and 15) and their cold gallium-complexes. Siderophores 7 and 15 were later radiolabeled with gallium-68, named as [^68^Ga]Ga-7 and [^68^Ga]Ga-15, respectively. Reproduced from Reference (240) with permission under CC BY-NC-ND 4.0. Reproduced with BioRender.com.

A recent study has synthesized a panel of 11 siderophores based on tetrapodal 1,4,7,10-tetraazacyclododecane-1,4,7,10-tetraacetic amide (DOTAM) and evaluated their potential for imaging infection (240). *In vitro* and *in vivo* characterization of these synthetic siderophores resulted in two potential radiotracers, [^68^Ga]Ga-7 and [^68^Ga]Ga-15 (Figure 9). A murine infection model using *E. coli* demonstrated both tracer’s ability to differentiate between muscle infection and LPS-mediated sterile inflammation. Design of tailored, synthetic siderophores may prove to be a promising future application for infection diagnostic purposes and may add valuable information to the current standard infection diagnostic techniques in use, as a combination approach improving sensitivity and specificity of pathogen detection.

Additionally, pathogen-specific tracers could also be used to specifically monitor the presence of viable bacteria and help in determining the treatment duration. This ^68^Ga-siderophore approach can be rationally combined with antibiotics or antimicrobials for treatment and management of infections (241). Once validated, these could also be utilized for precision medicine approaches for patients with complicated infections. Early and specific detection of infections as well as dual-tracer imaging approaches could provide accurate data on the class of bacteria causing the infections and potentially help in streamlining empirical antimicrobial therapies and reduce AMR burden.

No studies have explored the ability of ^68^Ga-complexes in microbial biofilm penetration. Synthetic and novel chelators based on siderophores targeting biofilm-specific molecules (from pathogens) could lead to the development of biofilm-specific imaging techniques, which might be particularly useful if bacteria within the biofilm are undetectable due to poor biofilm penetration of radiotracers, or due to bacterial quiescence.

Alternatively, radiotherapeutics for drug-resistant microbial infection by radiometals would be another strategy to address AMR. Although certain therapeutic radionuclides currently exist, evaluation of those radionuclides for antibacterial activity would be interesting. For example, ^67^Ga causes cytotoxicity and DNA damage in cancer cells that is comparable to ^111^In, and about 1Bq/cell is enough for complete killing in a non-specific targeted method (242). A pathogen-specific ^67^Ga-siderophore may enable systemic targeted radionuclide antimicrobial therapy, benefitting patients in whom antibiotic intolerance or resistance develops. This area of research is yet to be explored and requires several careful considerations including the radiation sensitivity of a range of microbial pathogens, radiocytotoxicity to surrounding human cells, and side-effects of such targeted antimicrobial therapy. Cold gallium has been shown to have antibacterial activity towards different microbes *in vitro* (243). Evaluation against clinically relevant pathogens must be performed to determine the minimum inhibitory radiation dose of ^67^Ga and/or ^67^Ga-siderophore combinations. Since some microorganisms can withstand high radiation doses (e.g., *E. coli* can tolerate ∼ 1 kGy) (244, 245) and normal tissue dose limits for human is minimum 2–5 Gy (depending on the organ type) (246), dosimetric considerations will be critical (247–249).

Hydroxypyridinones (HOPOs) are mainly used in iron chelation therapy. Synthetic derivation of HOPO multidentate bifunctional chelators have shown antimicrobial activity against *S. aureus*, *Bacillus subtilis*, *P. aeruginosa*, and *E. coli* in the preclinical settings (81). Thus, alternative antimicrobial treatment could be explored with synthetic siderophore therapy. Developing imaging probes for emerging pathogens like Mucorales, *Scedosporium apiospermum* complex and *C. auris* should be included in the near future. Invasive candidiasis should be targeted as no clinical probe for *C. albicans* based on ^68^Ga-siderophores is being investigated preclinically. Clinical respiratory isolates from CF patients of *Scedosporium apiospermum* complex are known to produce and excrete a siderophore, Nα-methyl coprogen B (250).

Additionally, ^68^Ga-labelling with modified TAFC, named DAFC, showed the proof of concept development of a theranostic (trojan-horse) approach by conjugating antifungal drugs with DAFC (251). *A. fumigatus* infection in lungs of a rat was monitored with gallium-68-labeled DAFC with several antifungal agents using PET/CT and the images were compared with control. The PET/CT images show differences in the distribution of radioactivity in these animal models suggesting possible effect of different antifungals on the infection. The prospective PET radiometals, Scandium-44 (^44^Sc) (half-life 3.9 h, generator produced) and Yttrium-86 (^86^Y) (half-life 147 h, cyclotron produced) could be explored for their new radiometal and chelation chemistry for infection imaging.

**Figure 10:**
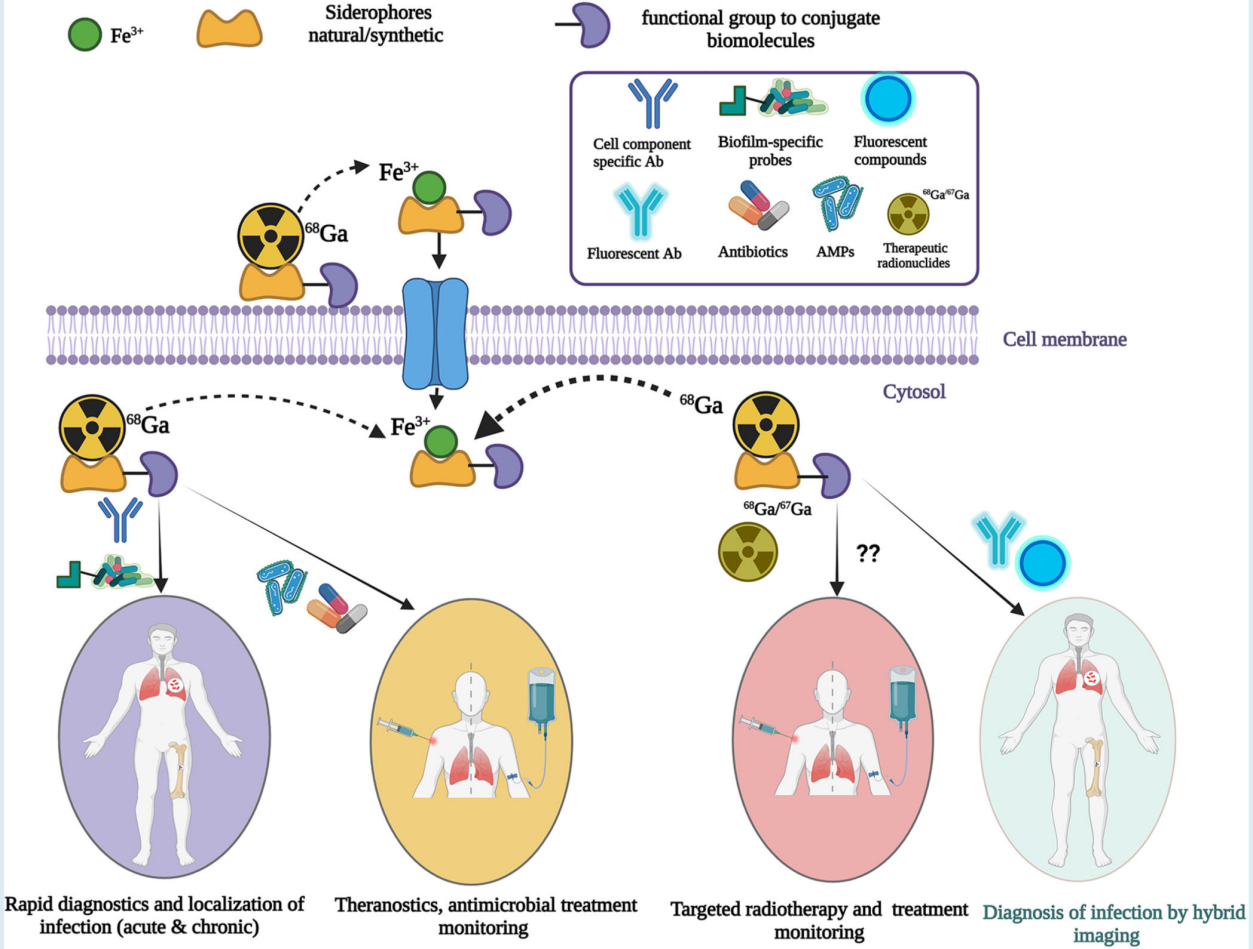
Prospects of ^68^Ga-labeled siderophores for infection diagnostics, targeted therapy and treatment monitoring. Dotted arrow indicates replacement of ferric ion by gallium-68. Theranostic approaches where diagnostics and treatments are combined. Double question marks indicates yet to explored approach with ^68^Ga-siderophores. Created with BioRender.com.

Figure 10 shows the prospects of ^68^Ga-siderophores for diagnostics and therapeutic (theranostic) purposes in microbial infection.

## 6 Conclusion

Bioconjugation of antibiotics and antimicrobial peptides with ^99m^Tc has been conducted in many preclinical infection models for infection imaging by SPECT. Clinical study with [^99m^Tc]Tc-UBI for knee prosthesis infection was able to detect infections in human. Later [^68^Ga]Ga-NOTA-UBI was able to diagnose bone and soft-tissue infection in all suspected patients.

Although multimodal *in vivo* imaging has great potential for the diagnosis of life threatening and difficult to treat microbial infections, available clinical imaging lacks the specificity to distinguish between microbial infection from cancer or sterile inflammation. Development of rapid imaging techniques to diagnose the presence of infection, the anatomical location of an infection, and the infecting pathogen, remains a prescient unmet need. Moreover, biofilms and persister cells add additional complexity to the detection and treatment of many infections. A targeted therapeutic approach (theranostics) would directly deliver antimicrobials to the site of infection and continuous monitoring of antimicrobial success/failure would complement the treatment process.

Considering exciting work on siderophore-based PET tracer development for infection imaging, it is envisaged that further studies exploring pathogen-specific siderophores (natural/modified/synthetic) could lead to development of PET tracers that are able to differentiate between bacterial and fungal infection and to distinguish between classes and/or species of bacterial and fungal pathogens. This could potentially supplement the diagnostic capabilities of current standard serological, microbiological, and molecular tools used in infection diagnosis. Chelator development for ^89^Zr and ^64^Cu will also enrich the number of PET tracers for infection diagnostics. Moreover, novel chelators for PET radiometals such as ^44^Sc and ^86^Y could develop new infection-specific tracers *via* bioconjugation of peptides and antibodies, and based on their PK/PD could be further developed for specific type of infection (e.g., location of infection, type of pathogens).

Since the use of radioactive gallium-labeled siderophores for microbial infection imaging has not been translated into the clinic yet, and this approach can lead to development of antimicrobial treatment monitoring tools using PET/CT and a vector for targeted antimicrobial drug/radiotherapy in high-risk patients, extensive preclinical work should be carried out to develop broad-spectrum and specific PET tracers. Due to its expensive nature, PET/CT is most suitable for nosocomial and high consequence infections, mainly in immunocompromised patients or in prosthetic infection. Guidelines for the preclinical and clinical assessment of novel imaging techniques should be established to facilitate the swift, safe, and appropriate clinical translation of new imaging techniques.

## Supplementary Material

Supplementary Figure S1

## Figures and Tables

**Table 1:** Common microbial pathogens that need to be addressed.

Bacterial Pathogens		
Gram-positive	Gram-negative	Others
*Staphylococcus aureus*	*Escherichia coli*	*Mycobacterium tuberculosis*
CoNS staphylococci (*S. epidermidis*, *S. hominis, etc.*)	*Pseudomonas aeruginosa*	
*Enterococcus*	*Klebsiella pneuomoniae*	
*Streptococcus pneumoniae*	*Acinetobacter baumannii*	
Fungal pathogens		
Molds	Yeasts	
*Aspergillus fumigatus*	*Candida albicans*	
*Mucoromycetes*	*C. parapsilosis*	
	*C. glabrata*	
	*C. auris*	

**Table 2:** PET and SPECT compatible radiometals and matching chelators for potential clinical application (78–84).

Radiometal	Half-life	Production method	Chelator	Imaging modality
^99m^Tc	6.01 h	^99^Mo/^99m^Tc Generator	Hexamethylpropyleneamine oxime (**HMPAO**), diethylenetriamine pentaacetate (**DTPA**), dimercapto succinic acid (**DMSA**), mercaptoacetyltriglycine (**MAG-3**), ethyl cysteinate dime (**ECD**), hydrazinonicotinamide (**HYNIC**)	SPECT
^111^In	67.4 h	Cyclotron, ^111^Cd(p, n)^lllm,g^In Cyclotron, ^112^Cd(p,2n)^111m,g^In	Oxine, **DTPA**, 1,4,7,10-tetraazacyclododecanetetraacetic acid (**DOTA**), N,N’-bis(2-hydroxy-5-sulfobenzyl)ethylenediamine-N,N’-diacetic acid (**SHBED**), bipyridine-chelator (**BPCA**), 6,6'-((ethane-1,2-diylbis ((carboxymethyl)azanediyl))bis-(methylene))dipicolinic acid (**H_2_octapa**)	SPECT
^67^Ga	78.3 h	Cyclotron, ^68^Zn(p,2n)^67^Ga	1,4,7-triazacyclononanetriacetic acid (**NOTA**), desferrioxamine (**DFO**)/desferrioxamine B (**DFO-B**), tris (hydroxypyridinone) (**THP**), 1,4,7-triazacyclononane phosphinic acid (**TRAP**), N,N'-bis(2-hydroxybenzyl)ethylenediamine-N,N'-diacetic acid (**HBED**), **SHBED**, tris-3,4-HP (**CP256**), 3,6,9,15-tetraazabicyclo[9.3.1]pentadeca-1(15),11,13-triene-3,6,9,-triacetic acid (**PCTA**), 1,2-[[6-(carboxy)-pyridin-2-yl]methylamino]ethane (**H_2_dedpa**)	SPECT
^64^Cu	12.7 h	Cyclotron, ^64^Ni(p,n)^64^Cu	**DOTA**, 1,4,8,11-tetraazacyclotetradecane-1,4,8,11-tetraacetic acid (**TETA**), 4,11-bis (carboxymethyl)-1,4,8,11-tetraazabicyclo[6.6.2]hexadecane (**CB-TE2A**), Diamsar, **NOTA**, **PCTA**, **H_2_dedpa**, N,N'-[1-benzyl-1,2,3-triazole-4-yl]methyl-N,N'-[6-(carboxy)pyridin-2-yl]-1,2-diaminoethane (**H_2_azapa**)	PET
^68^Ga	67.8 min	^68^Ge/^68^Ga Generator	**DOTA, DTPA, THP, DFO, NOTA, TRAP, HBED, SHBED, CP256, PCTA, H_2_dedpa**, 1,4,7-triazacyclononane (tacn) macrocycles substituted with phosphonic (**NOTP**)	PET
^89^Zr	78.41 h	Cyclotron, ^89^Y(p,n)^89^Zr	**DFO**, N,N'-(methylenephosphonate)-N,N'-[6-(methoxycarbonyl)pyridin-2-yl]methyl-1,2-diaminoethane (**H_6_phospa**), **HOPO**, **CP256**	PET
^44^Sc	3.9 h	^44^Ti/^44^Sc Generator	**DOTA, DTPA**	PET
^52g^Mn	5.5 days	Cyclotron, ^52^Cr(p,n)^52^Mn	**DOTA, PCTA, NOTA**	PET
^82^Rb	1.3 min	^82^Sr/^82^Rb Generator		PET
^86^Y	14.7 h	Cyclotron, ^86^Sr(p,n)^86^Y	**DTPA**, **DOTA**, 2,20-((2-(4,7-bis(carboxymethyl))-1,4,7-triazonan-1-yl)ethyl)azanediyl) diacetic acid (**NETA**)	PET

**Table 3:** Radionuclides and/or chelators for current clinical diagnostics of microbial infection.

Metal and/or chelators	Non-metals	Targeted infection/diseases	Limitation	References
[^99m^Tc]Tc-HMPAO/[^99m^Tc]Tc-exametazine		Osteomyelitis and spondylodiscitis, FUO, joint prosthesis, soft tissue infection, cardiovascular system infections, vascular prostheses, peripheral musculoskeletal infections	Non-specific accumulation in kidneys and intestines released by [^99m^Tc]Tc-exametazine	(23, 88–91)
[^111^In]In-oxine		Joint prosthesis and other orthopedic prostheses, imaging kidneys, bladder, gallbladder, and intestines	Non-specific, higher radiation dose	(23, 92)
[^67^Ga]Ga-citrate		Soft tissue infection, chronic osteomyelitis, opportunistic respiratory tract infections, FUO, tuberculosis, bone infection	Non-specific, higher radiation dose	(23, 89, 93–96)
[^68^Ga]Ga-citrate		Osteomyelitis, intra-abdominal infection, tuberculosis, interstitial nephritis, bacteremia, lung infection	Non-specific	(96–99)
	[^18^F] fluorodeoxyglucose	FUO, prosthetic vascular graft, ED, VGI, osteomyelitis, prosthetic joint infection, and musculoskeletal infections, bacteremia, fungal infection, mycobacterial infection	Non-specific	(20–22, 74, 99–104)

**Table 4:** Siderophores (endogenous and exogenous) in selected pathogens.

Pathogens	Native Siderophores	Siderophore receptor (OMR)	Xenosiderophores	References
*Pseudomonas aeruginosa*	Pyoverdine, Pyochelin	FpvA, FptA	Enterobactin, ferrioxamine and ferrichrome, mycobactin and carboxymycobactin, rhizobactin, aerobactin, schizokinen, vibriobactin	(152–154)
*Staphylococcus aureus*	Staphyloferrin A, Staphyloferrin B, Staphylopine, Aureochelin	HtsA SirA CntA ND	DFO-B, ferrichrome, aerobatin, coprogen, enterobactin, rhodorulic acid, baillibactin, salmochelin	(155–157)
*Klebsiella pneumoniae*	Aerobactin Yersiniabactin Enterobactin Salmochelin	IutA FyuA FepA IroN	-	(158, 159)
*Acinetobacter baumannii*	Acinetobactin Fimsbactin Baumannoferrins	BauA ND	-	(160, 161)
*Escherichia coli*	Enterobactin Salmochelin Aerobactin Yersiniabactin	FepA IroN IutA FyuA	-	(162)
*S. epidermidis*	Staphyloferrin A, Staphyloferrin B	HtsA SirA	DFO-B	(163, 164)
*Mycobacterium tuberculosis*	Mycobactin, Carboxymycobactin	ND	–	(165, 166)
*Aspergillus fumigatus*	Fusarinine C, Triacetylfusarinine C, Ferricrocin	MirB	(147)
*Candida albicans*	-	-	Ferrichrome, ferricrocin, ferrichrysin, ferrirubin, ferrioxamine B, ferrioxamine E	(167, 168)
*Mucorales*	Rhizoferrin	FslE	DFO-B	(169)

**Table 5:** ^68^Ga-labeled siderophores (endogenous and exogenous) investigated in different pathogens of interests.

^68^Ga-Siderophore	Microorganisms	*In vitro* up take	Stability in human serum/protein binding	*In vivo* clearance	Human lung cancer cell	Infection model	Reference
Pyoverdine (**PVD-POA1**)	*P. aeruginosa*	High and rapid	High stability	Renal excretion	No uptake	Myositis and lung infection	(181)
	*A. baumannii* *Burkholderia* *cenocepacia* *B. multivorans* *C. albicans* *E. coli* ATCC 10536 *K. pneumonia* *P.aeruginosa* ATCC 15692 *P. grimontii* *P. monteilii* *S.aureus* *S. maltophilia* *S. agalactiae* *Y. enterocolitica* *A. fumigatus* ATCC 46645	No				ND	(181)
Ornibactin (**ORNB**) Triacetylfusarinine C (**TAFC**) Ferrioxamine E (**FOXE**) Ferrichrome A (**FCHA**) **Aerobactin** (**AERO**)	*P. aeruginosa*	Negligible	ND	ND	ND	ND	(181)
**Yersiniabactin (YbT)**	*E. coli Nissle* 1917	High	?	?	?	Myositis (?)	(189)
	*E. coli* DHalpha	No	ND	ND	ND	ND	(189)
Desferrioxamine-B (**DFO-B**) derivatives	*S. aureus*	High	high stability	Renal excretion	ND	Myositis	(180)
**DFO-B**	*S aureus*	Highest and rapid	high stability ~ 20% protein binding up to 120 min incubation	Renal excretion	ND	Myositis	(183, 184)
	*P. aeruginosa*	High and rapid				Myositis & lung infectioin	
	*S. agalactia*	Higher and rapid				No	
	*A. fumigatus*	High at pH 7.0				Aspergillosis	
	*E. coli* ATCC 10536, *C. albicans* *K. pneumoniae*	No				ND	
**TAFC**	*A. fumigatus* ATCC46645	High and rapid	High	Renal excretion	ND	Neutronic IPA	(175, 178, 179)
	*A. terreus* DSM826, *A. flavus* ATCC 9643,	No				ND	
	*Rhizopus oryzae* AS5 *Fusarium solani* AS94	Low				ND	
	*C. albicans* ATCC 90028, *K. pneumoniae* *P. aeruginosa* ATCC 9027, *M. smegmatis* mc2155	No				ND	
	*S. aureus*	No				Abscess model	
**FOXE**	*A. fumigatus* ATCC46645	High and rapid	High	Renal excretion	ND	Neutronic IPA	
	*A. terreus* DSM826 *A. flavus* ATCC 9643, *R. oryzae* AS5 F. solani AS94	Low				ND	
	*C. albicans* ATCC 90028 *K. pneumoniae* *P. aeruginosa* ATCC 9027, *M. smegmatis* mc2155	No				ND	
	*S. aureus*	Low				Abscess model	
Ferricrocin (**FC**)	*A. fumigatus* ATCC 46645	High and rapid	60% protein binding	Blood & major organ retention	ND	ND	(176)
Coprogen (**COP**)	*A. fumigatus* ATCC 46645	Very low	Moderate	ND	ND	ND	(176)
Ferrichrome (**FCH**)	*A. fumigatus* ATCC 46645	High and rapid	60%	Blood&major organ retention	ND	ND	(176)
Ferrioxamine B (**FOXB**)	*A. fumigatus* ATCC 46645	Very low	10% protein binding	ND	ND	ND	(176)
Fusarinine C (**FUSC**)	*A. fumigatus* ATCC 46645	High and rapid	20% protein binding	High Kidney retention	ND	ND	(176)

ND, not determined/not done;?, not revealed in the publication.
