# Supplementary material for: Radiometal chelators for infection diagnostics
Source: Front Nucl Med. 2023 Jan 9;2:1058388. doi: 10.3389/fnume.2022.1058388 (PMC7614707; doi:10.3389/fnume.2022.1058388)
Supplement: Supplementary file 1 [file Datasheet1.docx]

Supplementary Material

**Supplementary Table S1.** Siderophore as Bi-functional chelators. Reproduced from Ref. (251) under Creative Commons Attribution 4.0 International License.

| **Radionuclide** | **Siderophore** | **Radiopharmaceutical** |
| --- | --- | --- |
| ^67^Ga | DFO | Albumin |
| ^67^Ga | DFO | Fibrinogen |
| ^67^Ga, ^111^In | DFO | Antibodies |
| ^68^Ga | DFO | Nanobodies |
| ^67^Ga | DFO | Folate |
| ^67^/^68^Ga | DFO | Octreotide |
| ^89^Zr | DFO | Antibodies |
| ^89^Zr | DFO | RGD peptides |
| ^89^Zr | DFO | Nanoparticles, carbon nanotubes, microspheres |
| ^89^Zr | DFO | Nanocolloids |
| ^89^Zr | DFO | Proteins |
| ^89^Zr | DFO* | Bombesins |
| ^68^Ga | FSC | Peptide multimers |
| ^89^Zr | FSC | Peptide multimers |

**Supplementary Table S2.** Summary of potential development in pathogen-specific radiotracer development (Promising radionuclides and/or chelators)

| **Chelators/ligand** | **Radiometal**  **/Radionuclides** | **Conjugate** | **Pathogen** | **Compound type-Targeted mechanism** | **Progress** |
| --- | --- | --- | --- | --- | --- |
|  | ^18^F | [^18^F]FDS | *E. coli, Klebsiella sp., Enterobacter sp., Salmonella sp., etc* | Sorbitol mechanisms | Clinical |
| - | ^99m^TC | UBI 18–35, UBI 31–38 fragment, UBI (29–41) fragment | *S. aureus, K pneumoniae, C. albicans* | Antimicrobial peptide- bacterial cell membrane | Preclinical¸ Clinical |
| NODAGA DOTA - NOTA | ^68^Ga | UBI (29–41) fragment | *S. aureus* | Antimicrobial peptide- bacterial cell membrane | Preclinical |
| NODAGA | ^64^Cu | hJF5 mAb | *A. fumigatus* | Antibody-fungal mannoprotein  antigen | Preclinical |
|  | ^18^F | [^18^F]F-trimethoprim | bacterial |  | Clinical |
|  | ^11^C | [^11^C]C-Trimethoprim | bacterial |  | Clinical |
|  | ^11^C-D-Met | D-[methyl-^11^C]methionine | *E. coli, S. aureus* |  | Clinical? |
| DFO-B(siderophores) | ^68^Ga | [^68^Ga]Ga-DFO-B | *P. aeruginosa*, *S. aureus* | Natural metal chelator- chelates essential iron (Fe III) | Clinical observation study |
| DFO-derivatives  (siderophores) | ^68^Ga | [^68^Ga]Ga-DFO -derivatives | *S. aureus* | Natural metal chelator- chelates essential iron (Fe III) | Preclinical |
| PVD  (siderophores) | ^68^Ga | [^68^Ga]Ga-PVD | *P. aeruginosa* | Natural metal chelator- chelates essential iron (Fe III) | Preclinical |
| TAFC, FOXE  (siderophores) | ^68^Ga | [^68^Ga]Ga-TAFC/FOXE | *A. fumigatus* |  | Preclinical |
| Yersiniabactin (YbT) (siderophore) | ^64^Cu | [^64^Cu]Cu-YbT | *E. coli*  *K. pneuniponiae* | Chelator for copper ions | Preclinical |


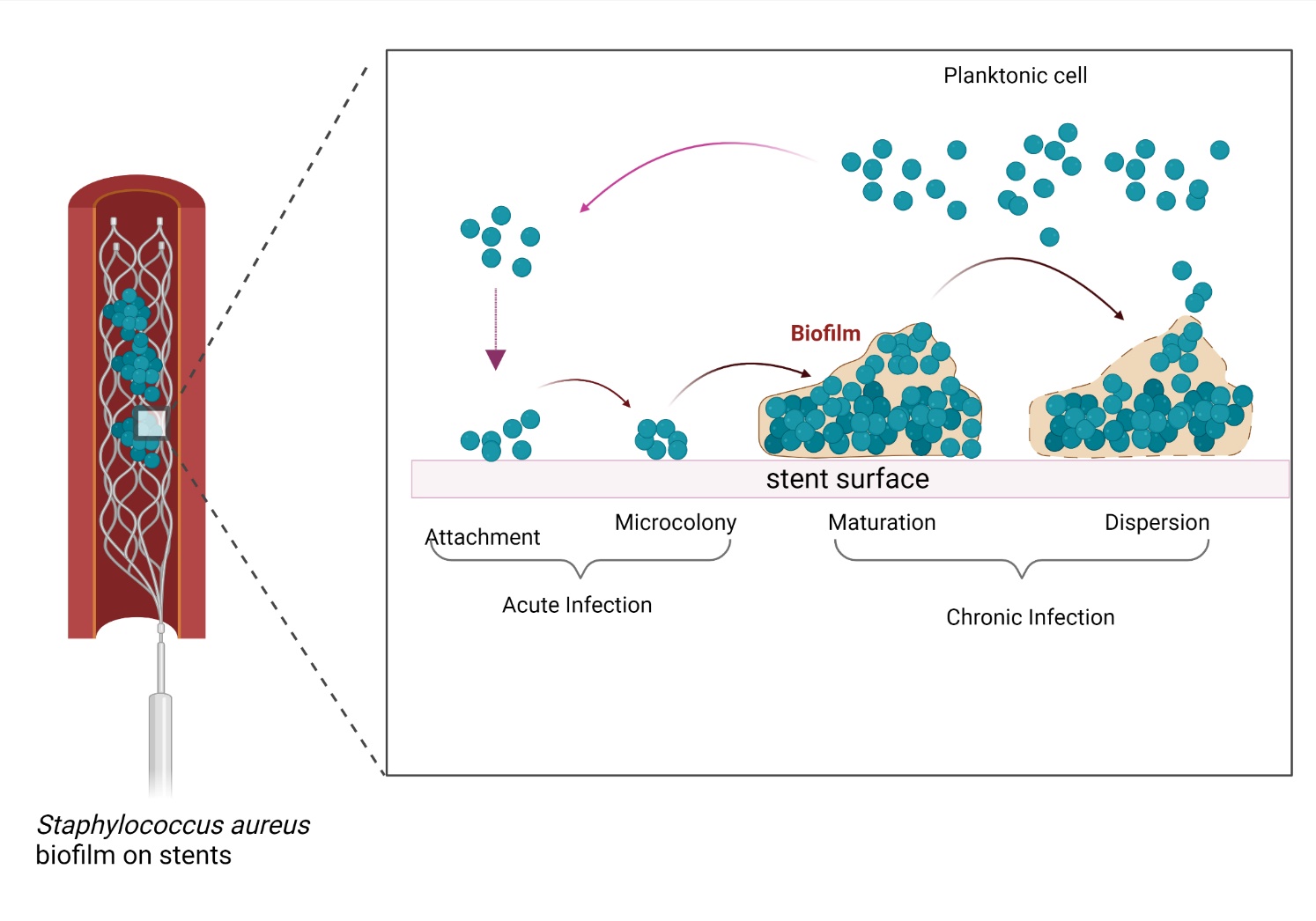


**Supplementary Figure S1.** Biofilm-infections in vascular stents by *S. aureus* strains. Created with BioRender.com


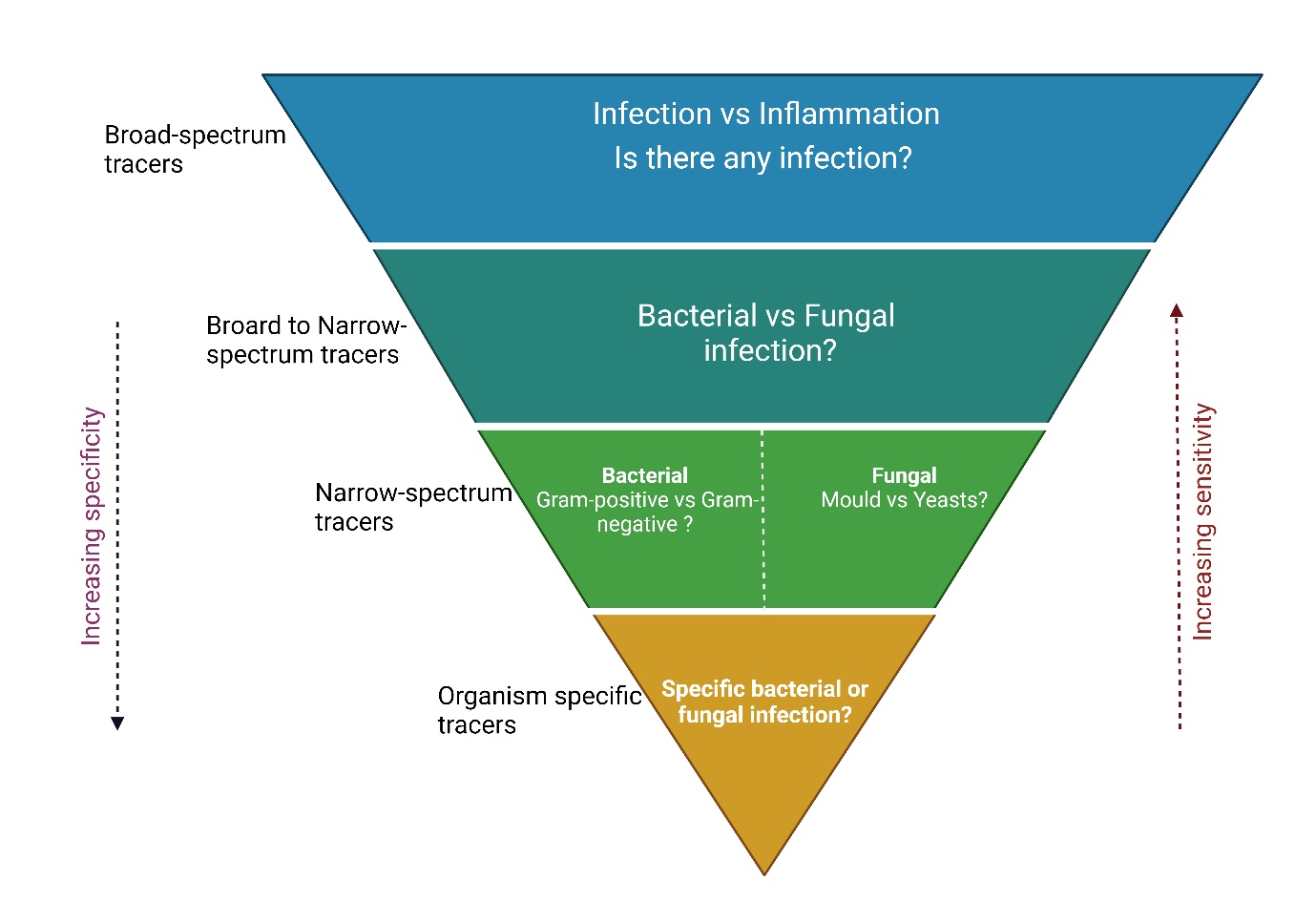


**Supplementary Figure S2.** Utility of broad- and narrow-spectrum radiotracer development for infection diagnostics. Created with BioRender.com
